# Supplementary material for: Cutaneous Immune Cell-Microbiota Interactions Are Controlled by Epidermal JunB/AP-1
Source: Cell Rep. 2019 Oct 22;29(4):844–859.e3. doi: 10.1016/j.celrep.2019.09.042 (PMC6856727; doi:10.1016/j.celrep.2019.09.042)
Supplement: Document S1. Figures S1–S7 and Table S1 [file mmc1.pdf]

Cell Reports, Volume *29*

Supplemental Information

Cutaneous Immune Cell-Microbiota Interactions Are Controlled by Epidermal JunB/AP-1

Özge Uluçkan, Maria Jiménez, Ben Roediger, Jakob Schnabl, Lucía T. Díez-Córdova, Kevin Troulé, Wolfgang Weninger, and Erwin F. Wagner

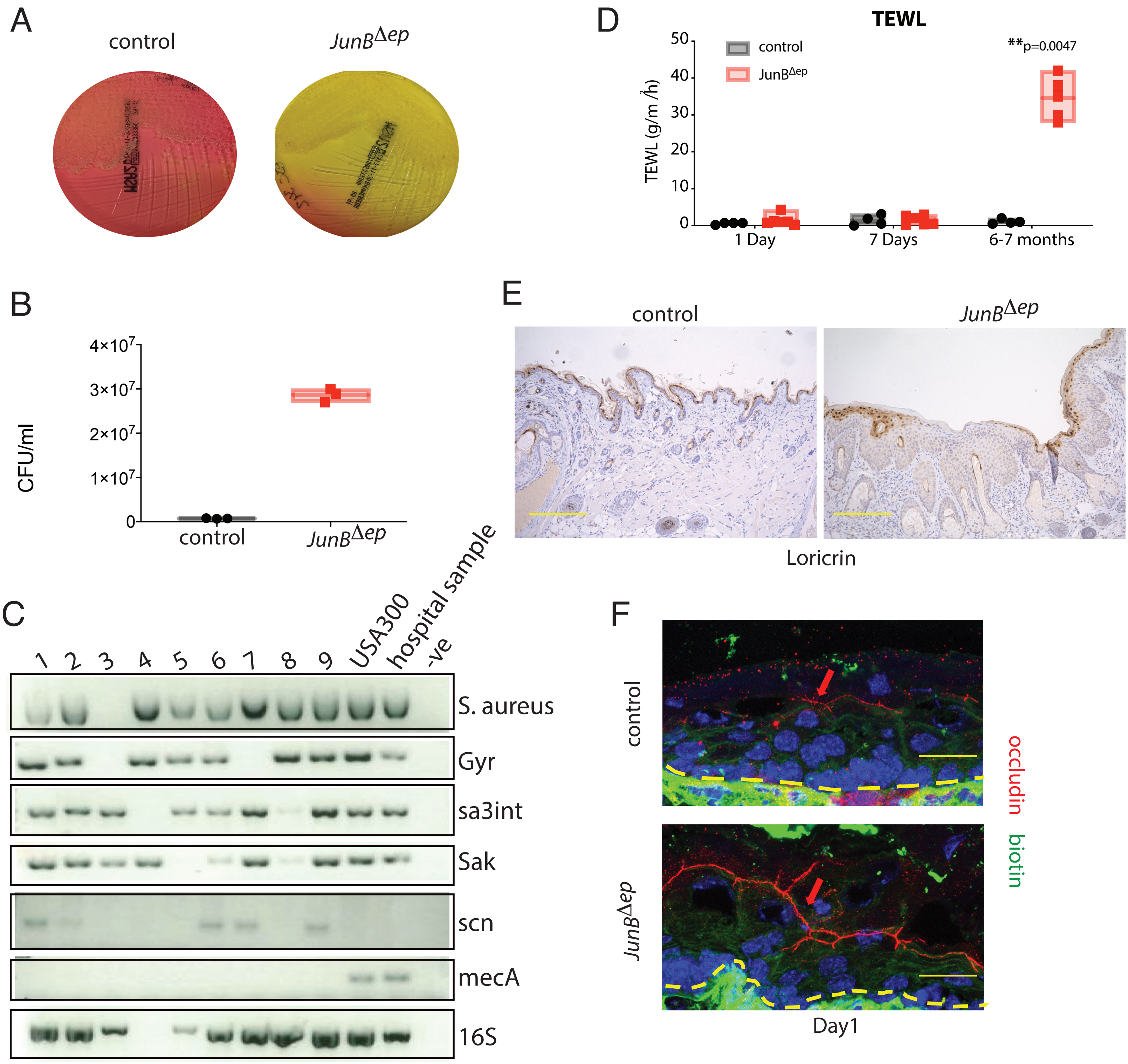

Figure S1. *JunB*$^{\Delta ep}$ mice have human-derived *S. aureus* colonization and transient barrier defects. Related to Figure 1. A) Growth of bacteria on mannitol-salt agar plates isolated from control vs *JunB*$^{\Delta ep}$ skin (n>10 per genotype). B) CFUs/ml of bacteria isolated control versus *JunB*$^{\Delta ep}$skin. C) PCR for S. aureus, S. aureus specific gyrase (gyr), human-specific immune invasion gene cluster including Staphylokinase (sak) and staphylococcal complement inhibitor (scn), a human-specific prophase integrase-gene sa3int and methicillin-resistance gene mecA with 16S as control from from single colonies of bacteria grown on blood agar plates from *JunB*$^{\Delta ep}$ skin. D) Trans-epidermal water loss measurements 1 and 7 days post-birth of control and *JunB*$^{\Delta ep}$ mice, as well as 6-7 months old control and *JunB*$^{\Delta ep}$ mice (n=4,6) (lesional skin). E) Loricrin staining at 6-7 months of age of the lesional skin of control and *JunB*$^{\Delta ep}$ mice (n=5,6). Yellow scale bars indicate 100µm. F) Inside-out barrier assay using biotin diffusion in control and *JunB*$^{\Delta ep}$ mice at 1-day post birth (n=4,5). Biotin-streptavidin is shown in green and occludin IF is shown in red.
Dashed yellow line indicates the basement membrane separating epidermis-dermis.
Red arrows show where the green biotin staining ends. Yellow scale bars indicate 50µm.

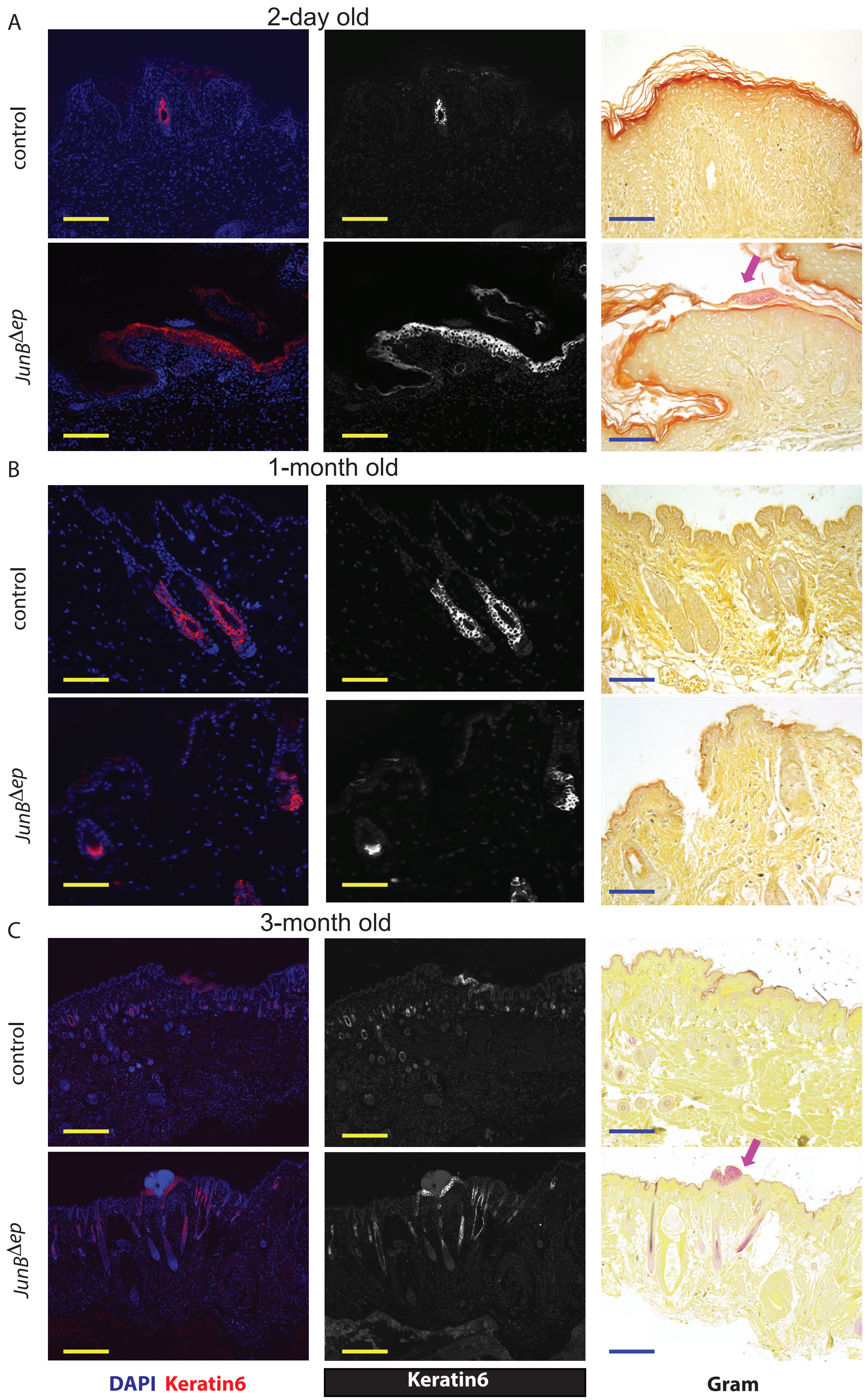

Figure S2. *JunB*$^{\Delta ep}$ mice do not have S. aureus colonization early in life. Related to Figure 1. Keratin6 and Gram staining in skin from control and *JunB*$^{\Delta ep}$ mice at 2 days (n=4,5), 1 month (n=4,4) and 3 months (n=7,8) post-birth. The yellow scale bars indicate 200μm in A, 100μm B and 500μm in C. The blue scale bars indicate 100μm in A, B and 500μm in C.

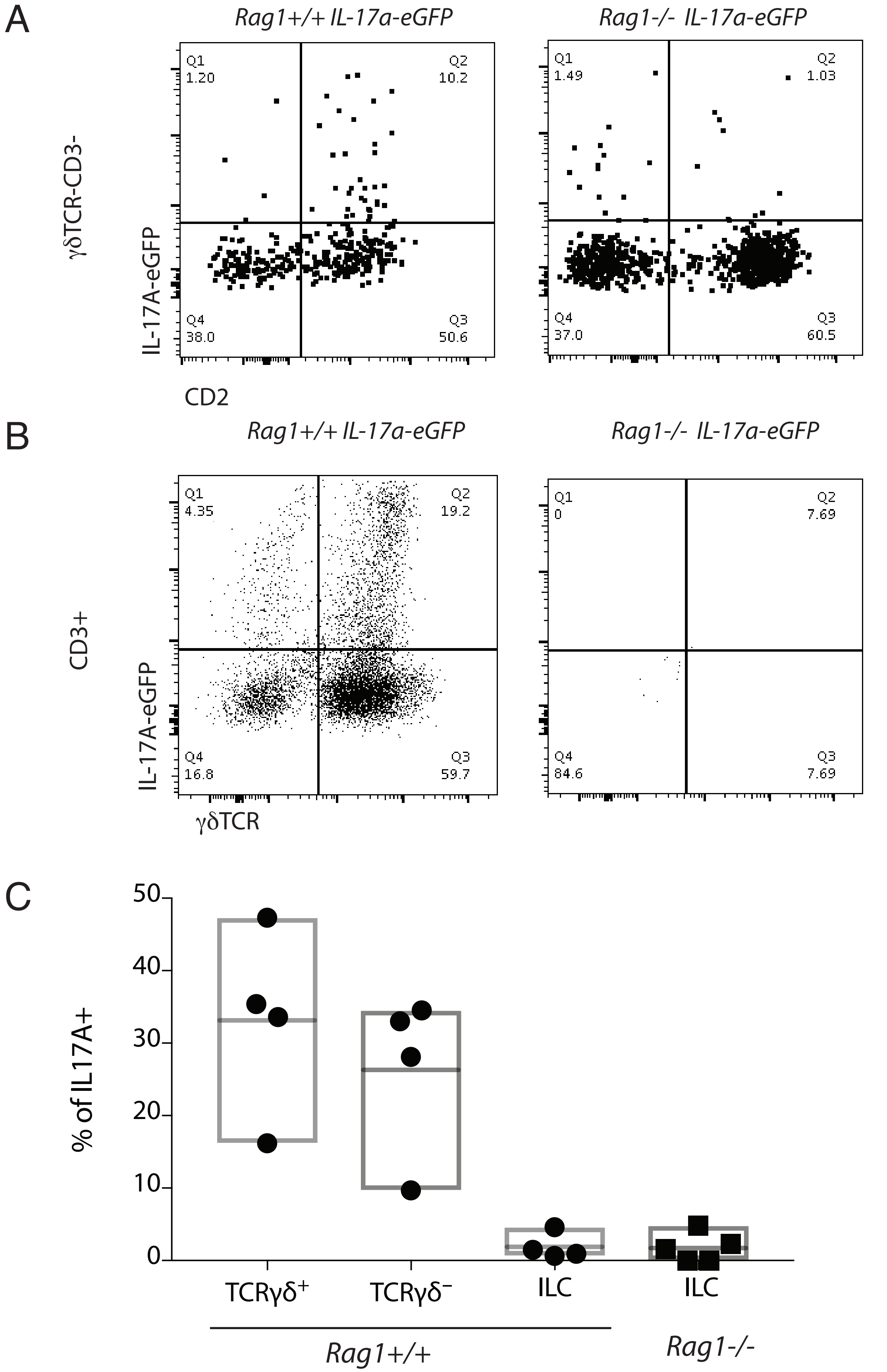

Figure S3. ILC3s do not compensate for IL-17A production by T-cells in *Rag1-/-* mice upon IMQ administration. Related to Figure 4.

A) Representative flow cytometry images of IL-17A expressing ILCs of control and *Rag1-/-* mice upon 5-days of IMQ administration on the ears (n=4,5). B) Representative flow cytometry images of IL-17A expressing T-cells of control and *Rag1-/-* mice 5-days of IMQ administration on the ears. C) Quantification of the distribution of IL-17A+ cells in *Rag1+/+* compared to *Rag1-/-* mice.

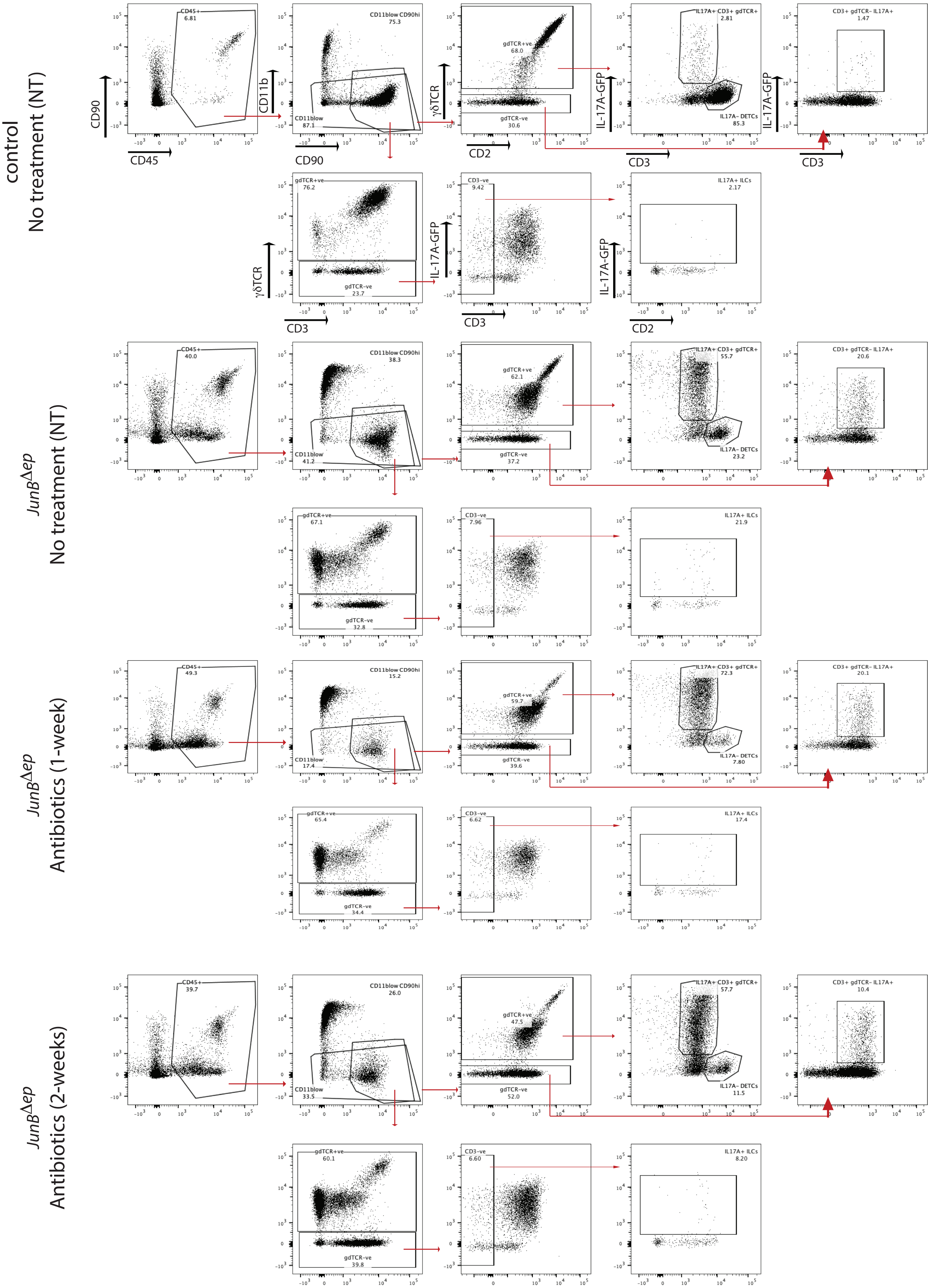

Figure S4. Antibiotic treatment post development of skin inflammation does not provide therapeutic benefit.
Related to Figure 6.
Conventional flow plots (including gating strategy) of lymphoid cells in *JunB*$^{\Delta ep}$ mice with or without 1 and 2-weeks of antibiotics compared to controls.

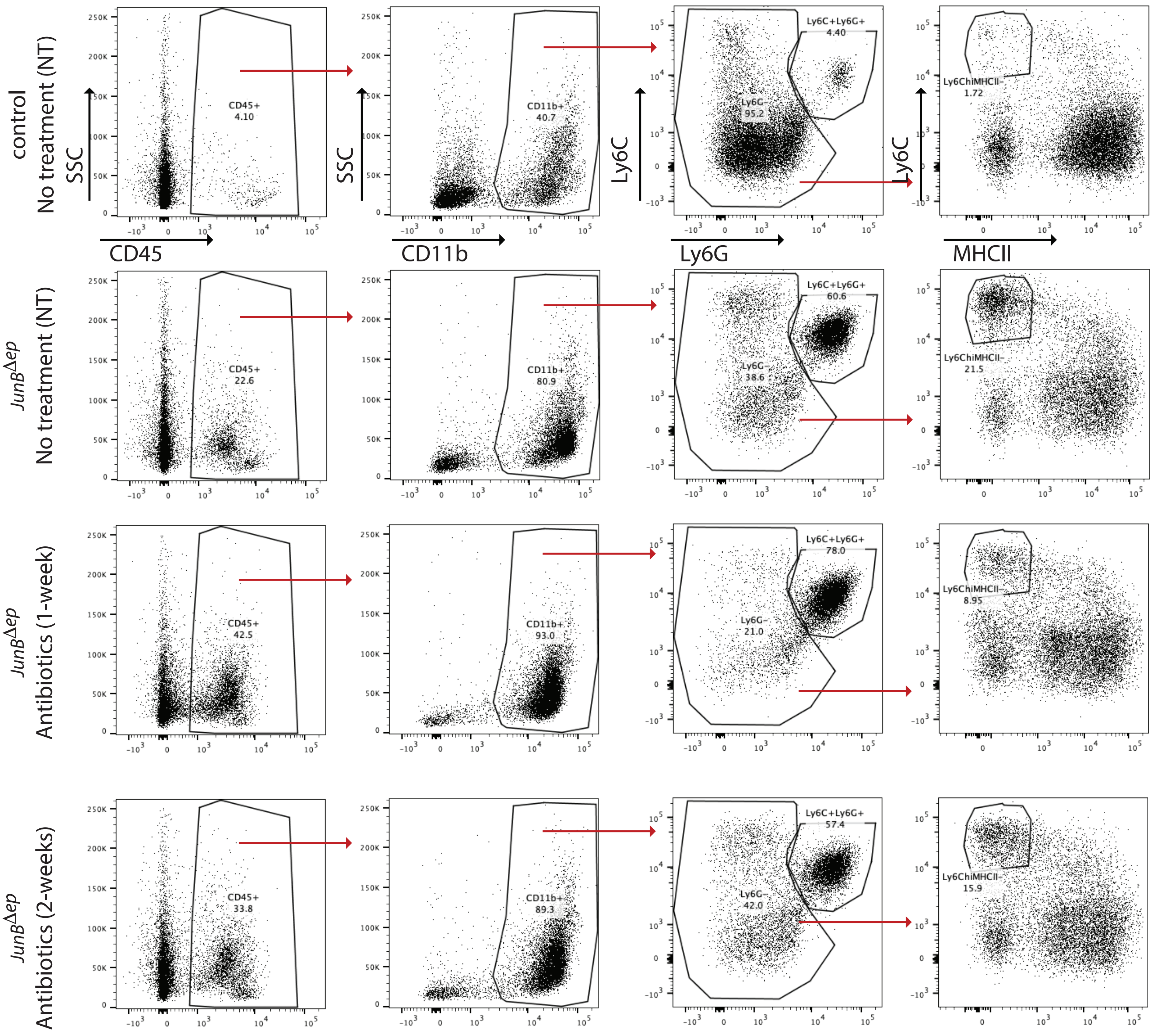

Figure S5. Antibiotic treatment post development of skin inflammation does not provide therapeutic benefit. Related to Figure 6.

Conventional flow plots (including gating strategy) of myeloid cells in *JunB*$^{\Delta ep}$ mice with or without 1 and 2-weeks of antibiotics compared to controls.

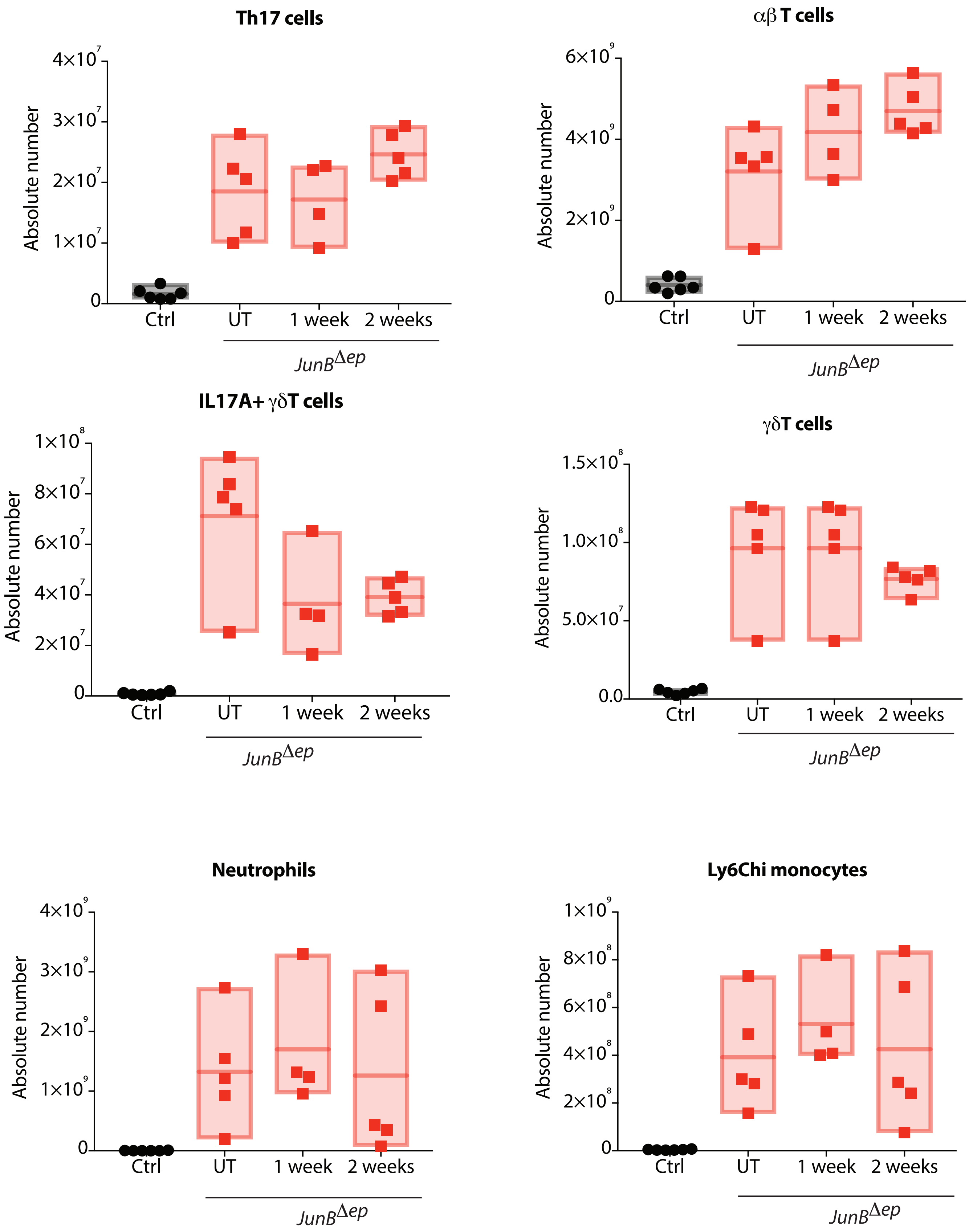

Figure S6. Increased number of γδTcells in lymph nodes upon 2-weeks of antibiotic treatment. Related to Figure 6. Number of infiltrating immune cells into the skin-draining lymph nodes of *JunB*$^{\Delta ep}$ mice with or without 1 and 2-weeks of antibiotics compared to controls as analysed by flow cytometry.

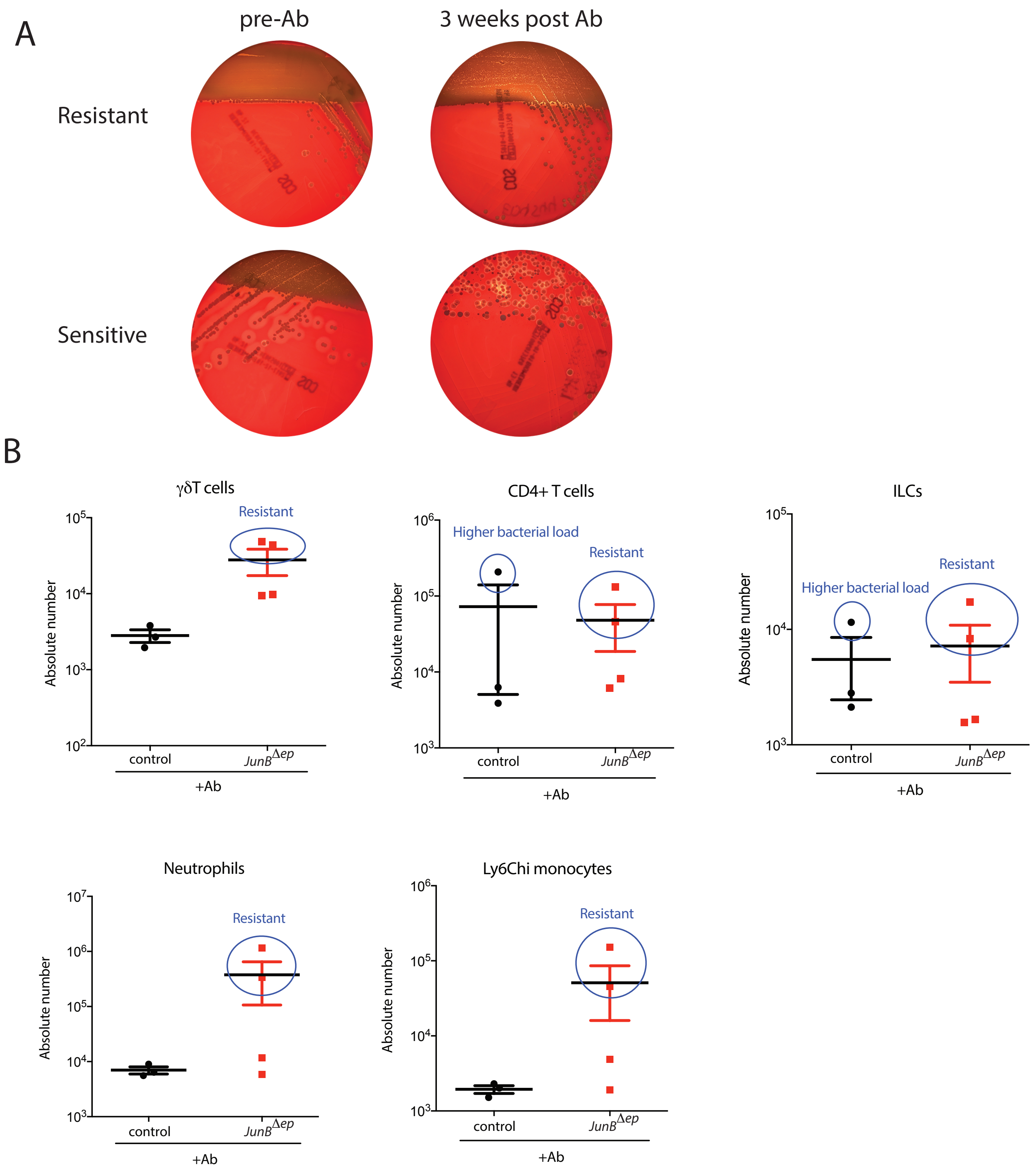

Figure S7. Characterization of the immune cells in mice resistant to antibiotics. Related to Figure 6.
A) Representative images of blood agar plates of swabs derived from *JunB*$^{\Delta ep}$ mice skin sensitive or resistant to 3-weeks of antibiotic treatment post-lesion development.
B) Number of immune cell infiltrates into the skin of *JunB*$^{\Delta ep}$ mice skin sensitive or resistant to 3-weeks of antibiotic treatment post-lesion development

Name	F (5'-3')	R (5'-3')
**Duox1**	GATTCTCCTGGTTGGGACA	CCTGCAGAACCTCTCATGA
**Il13ra2**	AGCGAATGGAGTGAAGAGGA	GCTCAATGTGGGTTCAGGTT
**Stac2**	CAAGCTCCAGCGATTTAAGCG	AGCCTCACTGGTTTGAGCG
**Tslp**	ACGGATGGGGCTAACTTACAA	AGTCCTCGATTTGCTCGAACT
**Aqp3**	AACCCTGCTGTGACCTTTG	GCTGCTGTGCCTATGAACTG
**Il4ra**	TGGGCTGTCGATTTTGCTTTTGG	GTGCTGGGGTGGGAATCTGGTC
**Nod2**	CAGGTCTCCGAGAGGGTACTG	GCTACGGATGAGCCAAATGAAG
**Osm**	TGCCCGGCACAATATCCTC	GTGTGGGCTCAGGTATCTCCAG
**Slc4a11**	CAGGACTCCGGTGAATACTTCT	GATGCTCTCGCCAGACACAA
**Mmp13**	CTTCTTCTTGTTGAGCTGGACTC	CTGTGGAGGTCACTGTAGACT
**Serpinb3a**	CAGATGATGAAACAAAACATCG	AGACCTTGAGTGCTGCTCATA
**il4**	GGTCTCAACCCCCAGCTAGT	GCCGATGATCTCTCTCAAGTGAT
**il1b**	GCAACTGTTCCTGAACTCAACT	ATCTTTTGGGGTCCGTCAACT
**lcn2**	TGGCCCTGAGTGTCATGTG	CTCTTGTAGCTCATAGATGGTGC
**S100a8**	AAATCACCATGCCCTCTACAAG	CCCACTTTTATCACCATCGCAA
**mdefb14**	TCCAGGGGACGCATTCCTA	ACCGCTATTAGAACATCGACCTA
**il36a (il1f6)**	GCAGCATCACCTTCGCTTAGA	CAGATATTGGCATGGGAGCAAG
**il36b (il1f8)**	AGAGTATTCAAATGTGGGAACCG	GACCCATACCATCTGTTGTGAG
**il36g (il1f9)**	TCCTGACTTTGGGGAGGTTTT	TCACGCTGACTGGGGTTACT
**cxcl1**	CTGGGATTCACCTCAAGAACATC	CAGGGTCAAGGCAAGCCTC
**cxcl2**	CCAACCACCAGGCTACAGG	GCGTCACACTCAAGCTCTG
**ccl2**	TTAAAAACCTGGATCGGAACCAA	GCATTAGCTTCAGATTTACGGGT
**S100a9**	ATACTCTAGGAAGGAAGGACACC	TCCATGATGTCATTTATGAGGGC
**S.aureus**	AATCTTTGTCGGTACACGATATTCTTCACG	CGTAATGAGATTTCAGTAGATAATACAACA
**Gyr**	AGTACATCGTCGTATACTATATGG	ATCACGTAACAGTTCAAGTGTG
**sa3int**	GAAAAACAAACGGTGCTAT	TTATTGACTCTACAGGCTGA
**Sak**	AAGGCGATGACGCGAGTTAT	GCGCTTGGATCTAATTCAAC
**scn**	AGCACAAGCTTGCCAACATCG	TTAATATTTACTTTTTAGTGC
**mecA**	AAAATCGATGGTAAAGGTTGGC	AGTTCTGCAGTACCGGATTTGC
**16S**	9AACTCTGTTATTAGGGAAGAACA	CCACCTTCCTCCGGTTTGTCACC

Table S1. Sequences of Oligonucleotides. Related to STAR Methods.
