# Supplementary material for: Cutaneous Immune Cell-Microbiota Interactions Are Controlled by Epidermal JunB/AP-1
Source: Cell Rep. 2019 Oct 22;29(4):844–859.e3. doi: 10.1016/j.celrep.2019.09.042 (PMC6856727; doi:10.1016/j.celrep.2019.09.042)
Supplement: Document S2. Article plus Supplemental Information [file mmc2.pdf]

Cell Reports

Article

Cutaneous Immune Cell-Microbiota Interactions Are Controlled by Epidermal JunB/AP-1

Graphical Abstract

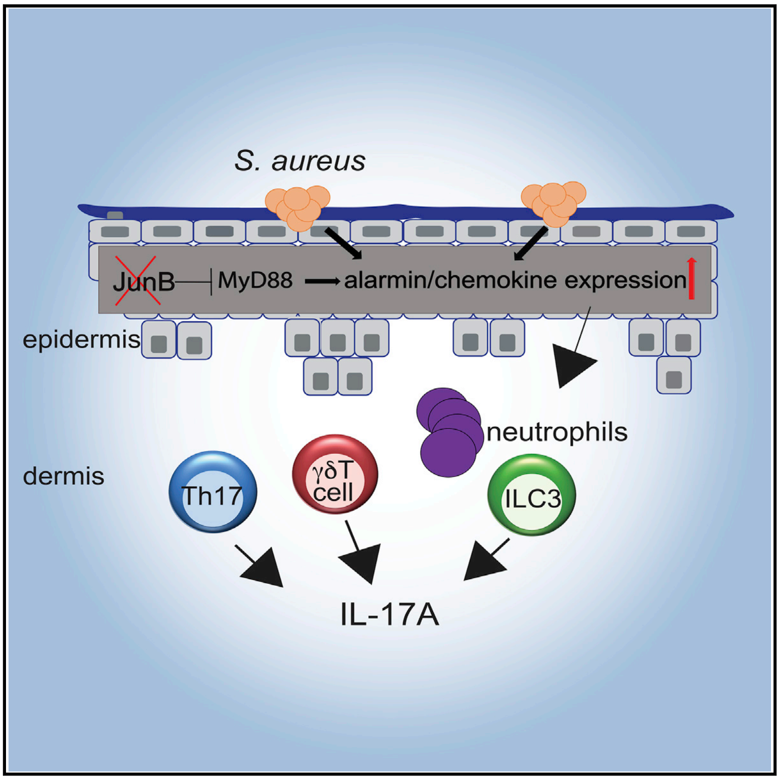

Highlights

- *JunB*$^{\Delta ep}$ mice display several defining features of atopic dermatitis
- Skin of *JunB*$^{\Delta ep}$ mice are colonized by human-derived *S. aureus*
- Adaptive immune system is necessary for protection against *S. aureus*
- JunB is an upstream regulator of the microbiota-immune cell interactions

Authors

Özge Uluçkan, Maria Jiménez, Ben Roediger, ..., Kevin Troulé, Wolfgang Weninger, Erwin F. Wagner

Correspondence

ozge.uluckan@novartis.com (Ö.U.), erwin.wagner@meduniwien.ac.at (E.F.W.)

In Brief

Atopic dermatitis is a complex, multi-factorial inflammatory skin disease. Uluçkan et al. show that *JunB*$^{\Delta ep}$ mice display several hallmarks of AD, including *S. aureus* colonization. The authors characterize the immune responses to *S. aureus* and show that the adaptive immune system is required for protection against *S. aureus* colonization.

Uluçkan et al., 2019, Cell Reports *29*, 844–859
October 22, 2019 © 2019 The Authors.
https://doi.org/10.1016/j.celrep.2019.09.042

OPEN ACCESS
CellPress

Cell Reports
Article

Cutaneous Immune Cell-Microbiota Interactions Are Controlled by Epidermal JunB/AP-1

Özge Uluçkan,[1,6,8,*] Maria Jiménez,[1] Ben Roediger,[2,6] Jakob Schnabl,[1,7] Lucía T. Díez-Córdova,[1] Kevin Troulé,[3] Wolfgang Weninger,[2,4] and Erwin F. Wagner[1,4,5,*]
[1]Cancer Cell Biology Program, Spanish National Cancer Research Centre (CNIO), 28029 Madrid, Spain
[2]Centenary Institute, Faculty of Medicine and Health, The University of Sydney, Camperdown, NSW 2050, Australia
[3]Bioinformatics Unit, Structural Biology and Biocomputing Programme, Spanish National Cancer Research Centre (CNIO), 28029 Madrid, Spain
[4]Department of Dermatology, Medical University of Vienna, Währinger Gürtel 18-20, 1090 Vienna, Austria
[5]Department of Dermatology and Department of Laboratory Medicine, Medical University of Vienna, Lazarettgasse 14a, 1090 Vienna, Austria
[6]Present address: Autoimmunity, Transplantation, Inflammation (ATI) Disease Area, Novartis Institutes for Biomedical Research, Basel 4056, Switzerland
[7]Present address: Institute of Molecular Biotechnology of the Austrian Academy of Sciences (IMBA), Vienna BioCenter (VBC), Dr. Bohrgasse 3, 1030 Vienna, Austria
[8]Lead Contact
*Correspondence: ozge.uluckan@novartis.com (Ö.U.), erwin.wagner@meduniwien.ac.at (E.F.W.)
https://doi.org/10.1016/j.celrep.2019.09.042

SUMMARY

Atopic dermatitis (AD) is a multi-factorial skin disease with a complex inflammatory signature including type 2 and type 17 activation. Although colonization by *S. aureus* is common in AD, the mechanisms rendering an organism prone to dysbiosis, and the role of IL-17A in the control of *S. aureus*-induced skin inflammation, are not well understood. Here, we show several pathological aspects of AD, including type 2/type 17 immune responses, elevated IgE, barrier dysfunction, pruritus, and importantly, spontaneous *S. aureus* colonization in $JunB^{\Delta ep}$ mice, with a large transcriptomic overlap with AD. Additionally, using $Rag1^{-/-}$ mice, we demonstrate that adaptive immune cells are necessary for protection against *S. aureus* colonization. Prophylactic antibiotics, but not antibiotics after established dysbiosis, reduce IL-17A expression and skin inflammation, examined using *Il17a-eGFP* reporter mice. Mechanistically, keratinocytes lacking JunB exhibit higher MyD88 levels *in vitro* and *in vivo*, previously shown to regulate *S. aureus* colonization. In conclusion, our data identify JunB as an upstream regulator of microbiota-immune cell interactions and characterize the IL-17A response upon spontaneous dysbiosis.

INTRODUCTION

Atopic dermatitis (AD) is a debilitating, chronic inflammatory skin disease affecting 15%–20% of children and up to 10% of adults (Weidinger et al., 2018). AD is characterized by epidermal hyperplasia, infiltration of innate and adaptive immune cells into the dermis, severe pruritus, and *S. aureus* colonization and superinfections (Geoghegan et al., 2018; Weidinger et al., 2018). The pathogenesis of AD is not well understood, although barrier defects, microbial dysbiosis, genetic predisposition, and environmental factors are thought to contribute to disease.

Transcriptomic analysis of AD patient biopsies has shown enrichment for type 2 immune pathways, the importance of which is highlighted by the efficacy of dupilumab, an IL-4Rα antibody that inhibits IL-4 and IL-13 signaling in AD patients (Simpson et al., 2016). However, higher levels of IL-17 and upregulation of the IL-17 signaling pathway have also been observed in AD (Koga et al., 2008; Werfel et al., 2016). Interestingly, transcriptomic analysis comparing pediatric and adult AD skin biopsies showed more dominant Th17 pathway activation in pediatric patients, which accompanied higher levels of IL-36 expression (Brunner et al., 2018). Whether the increase in IL-17 observed in AD is protective or pathological remains to be determined. In inflammatory bowel disease, IL-17A has been shown to be protective for the barrier (Uluçkan and Wagner, 2017), and animal studies similarly suggest that IL-17A and its receptor maintain the skin barrier function (Naik et al., 2015; Floudas et al., 2017). In addition, IL-17A is postulated to lead to increased anti-microbial production from epithelial cells, which is lower in AD compared with psoriasis (Uluçkan and Wagner, 2017). Consistent with this, recombinant IL-17A given at the time of *S. aureus* challenge rescues the increased inflammation observed in TCRγδ T cell-deficient mice, suggesting a protective role of IL-17A in *S. aureus*-induced skin inflammation (Cho et al., 2010). Furthermore, IL-17A-secreting dendritic cells interacting with the skin microbiota have been shown to be essential to protect the skin from *S. aureus* infections (Naik et al., 2015). These studies suggest a protective role of IL-17A signaling in *S. aureus* colonization, through effects of the commensal bacteria. In contrast, mice lacking IL-17A/F have reduced skin inflammation upon *S. aureus* challenge, implicating a complex role of IL-17A during dysbiosis (Liu et al., 2017; Nakagawa et al., 2017). Therefore, the role of IL-17 remains unclear in cutaneous dysbiosis and AD, and further studies are needed to better

 © 2019 The Authors.
This is an open access article under the CC BY license (http://creativecommons.org/licenses/by/4.0/).

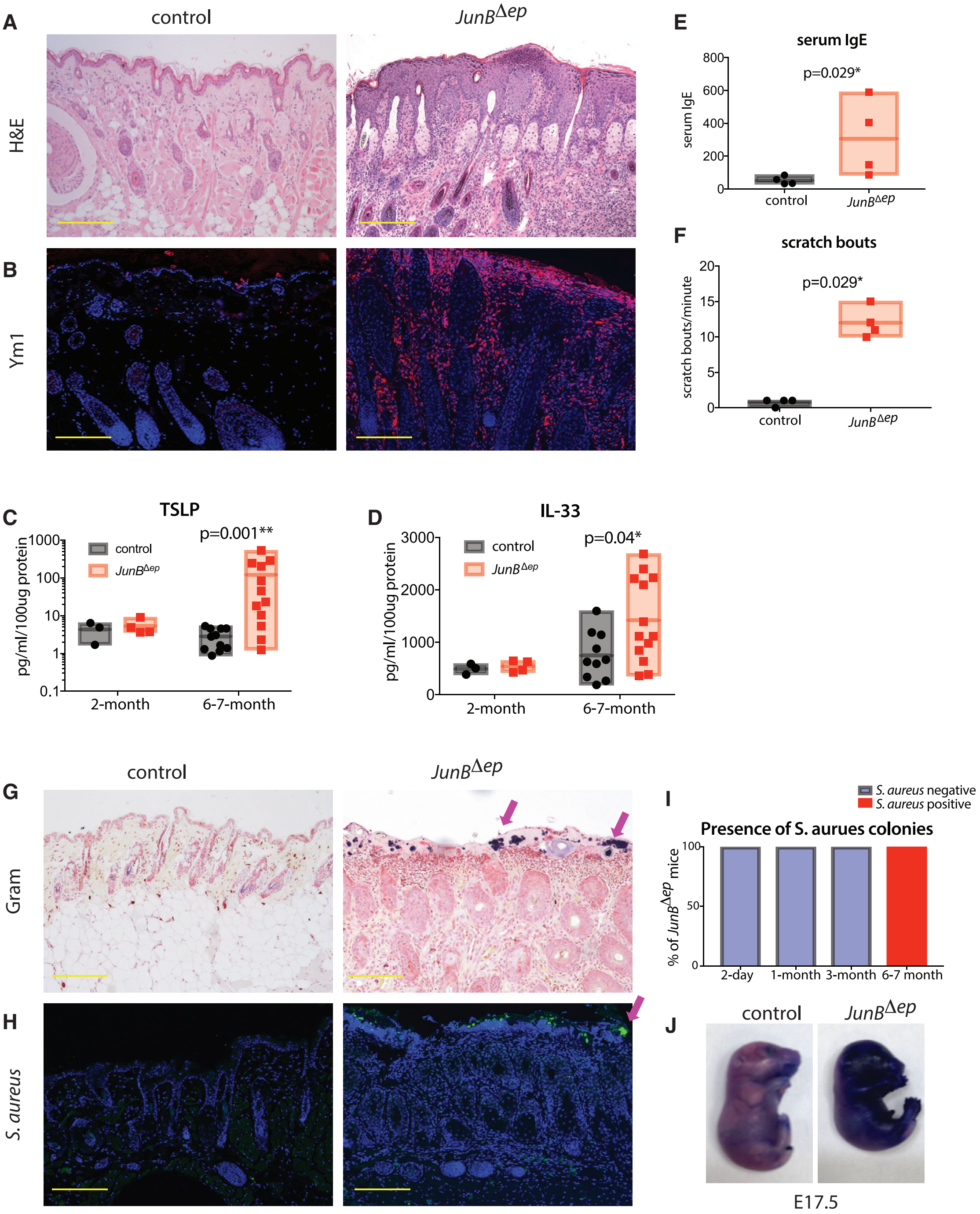

Figure 1. Spontaneous *S. aureus* Colonization with AD Features Is Observed in *JunB$^{\Delta ep}$* Mice

(A) Representative images of H&E staining of skin from control and *JunB$^{\Delta ep}$* mice at 6–7 months of age (n > 10 per genotype). The yellow scale bar indicates 200 μm.

(B) Representative images of Ym1 staining of skin from control and *JunB$^{\Delta ep}$* mice at 6–7 months (n = 4, 4).

(C) TSLP levels in skin lysates of control and *JunB$^{\Delta ep}$* mice at 2 (n = 3, 4) and 6–7 months (n = 11, 12).

(D) IL-33 levels in skin lysates of control and *JunB$^{\Delta ep}$* mice at 2 (n = 3, 4) and 6–7 months (n = 11, 12).

(E) Serum IgE levels of control and *JunB$^{\Delta ep}$* mice at 6–7 months of age (n = 4, 4).

(F) Scratch bouts of control and *JunB$^{\Delta ep}$* mice at 6–7 months of age (n = 4, 4).

(G) Representative images of Gram staining of skin from control and *JunB$^{\Delta ep}$* mice at 6–7 months (n > 10 per genotype). Arrows indicate Gram-positive colonies of bacteria. The yellow scale bar indicates 200 μm.

(legend continued on next page)

understand how this cytokine interacts with type 2 inflammation, barrier defects and the microbiota.

There is strong evidence to suggest that *S. aureus* exacerbates AD (Chen et al., 2018; Geoghegan et al., 2018). *S. aureus* colonization is observed at a higher frequency in lesional skin, and bacterial load correlates with disease severity (Gong et al., 2006). However, it remains unclear whether *S. aureus* colonization is a cause or a consequence of AD pathology. The heterogeneity of the skin microbiome, which influences *S. aureus* colonization, varies depending on the body site and further complicates investigations into the role of *S. aureus* in AD (Kong et al., 2012). Thus, the question of whether dysbiosis precedes *S. aureus* infection and type 2 immune activation or is secondary to barrier perturbation, resulting in type 2 immune activation, remains unresolved. A recent study showed that dysbiosis can be detected before the onset of inflammatory lesions in children, suggesting that *S. aureus* colonization might be causal to AD (Meylan et al., 2017).

The molecular mechanisms leading to *S. aureus* colonization and superinfections in AD patients remains poorly understood. Two recent studies identified an important role for MyD88 signaling in *S. aureus* colonization in mice (Liu et al., 2017; Nakagawa et al., 2017). However, whereas Liu et al. (2017) suggested that deletion of MyD88 in keratinocyte reduces disease burden, Nakagawa et al. (2017) concluded that MyD88 signaling in T cells is sufficient to control *S. aureus* colonization and skin inflammation. Both studies demonstrated IL-17A production downstream of *S. aureus* and that IL-17A/F double-knockout mice have reduced *S. aureus* colonization. These effects are attributed to the secretion of IL-1 family members from keratinocytes, particularly IL-36α. However, these studies are limited by the challenge method of *S. aureus* administration, which does not reflect the natural development of dysbiosis reported in AD. Indeed, a major impediment to our understanding of dysbiosis has been the paucity of mouse models with spontaneous *S. aureus* colonization. Mice lacking the membrane protease ADAM-17 are prone to cutaneous dysbiosis and AD-like skin inflammation, and Kobayashi et al. (2015) exploited this observation to demonstrate that *Corynebacterium bovis* induces type 2 inflammation, whereas *S. aureus* exacerbates disease. Nevertheless, several outstanding questions regarding the role keratinocytes and T cells in dysbiosis-induced inflammation and the regulation and cellular sources of IL-17A within the skin remain unsolved.

Here we show that mice with epidermal deletion of JunB/AP-1 exhibit spontaneous skin inflammation with several hallmarks of AD, including high IgE, barrier dysfunction, type 2 inflammation, pruritus, and, critically, spontaneous *S. aureus* colonization. Using this mouse model of cutaneous dysbiosis, we demonstrate a critical role for the adaptive immune system in preventing superinfections and investigate the effect of prophylactic and post-infection antibiotic treatment. Mechanistically, we explored the relationship between *S. aureus* colonization and IL-17A production within the skin and report an important role for JunB in regulating MyD88 expression within keratinocytes. Our data resolve outstanding questions about the role of *S. aureus* in eczematous inflammation and describe a model of spontaneous dysbiosis with chronic inflammation.

RESULTS

Spontaneous *S. aureus* Colonization with AD-like Features Is Observed in *JunB*$^{\Delta ep}$ Mice

We have previously shown that *JunB*$^{\Delta ep}$ mice, which have a deletion of *JunB* in K5-expressing cells, exhibit spontaneous chronic skin inflammation with concurrent bone loss through IL-17 signaling (Uluçkan and Wagner, 2016; Meixner et al., 2008; Pflegerl et al., 2009; Uluçkan et al., 2016; Singh et al., 2018). Upon further characterization of the skin inflammation of these mice, we observed spontaneous *S. aureus* colonization, a hallmark of AD. As mouse models of AD with spontaneous dysbiosis and clinical translatability are currently lacking (Jin et al., 2009; Ewald et al., 2017), we further investigated the pathophysiological characteristics of skin inflammation in these mice. *JunB*$^{\Delta ep}$ mice develop severe skin inflammation with hyperkeratosis and immune cell infiltration by 6 months of age (Figure 1A). We show that *JunB*$^{\Delta ep}$ skin has infiltration of Ym1-positive cells (Figure 1B), increased TSLP and IL-33 levels in skin (Figures 1C and 1D), elevated serum IgE (Pflegerl et al., 2009) (Figure 1E), and significant pruritus (Figure 1F), all important hallmarks of type 2 inflammation and AD (Welch et al., 2002; Nair et al., 2003; Bird, 2005; Cevikbas and Steinhoff, 2012; Doherty et al., 2013; Wilson et al., 2013; Turner and Zhou, 2014).

Additionally, Gram staining and immunofluorescence analysis using a specific *S. aureus* antibody showed extensive colonization by Gram-positive *S. aureus* on the skin surface of *JunB*$^{\Delta ep}$ mice (Figures 1G and 1H). We confirmed the identity of the Gram-positive bacteria to be *S. aureus* by growth and fermentation (yellow appearance) on mannitol-salt agar plates, a specific ability of *S. aureus* (Figure S1A), and measured colony-forming units (CFUs) of *S. aureus* (Figure S1B). We also isolated single colonies and carried out PCR with *S. aureus*-specific primers, as well as *S. aureus*-specific gyrase (*gyr*) (Figure S1C). At 6–7 months of age, 100% of *JunB*$^{\Delta ep}$ mice had *S. aureus* colonization and skin inflammation, whereas up to 3 months of age, no *S. aureus* colonization was observed (Figure 1I). To determine the kinetics of dysbiosis in these mice, we carried out Gram staining, coupled with immunofluorescence for keratin 6 as a marker of early inflammatory signals on keratinocytes at 2 days, 1 month, and 3 months post-birth. We observed sporadic Gram-negative colonies with surrounding keratinocytes positively stained for keratin 6, suggesting that prior to the colonization of *S. aureus*, some Gram-negative bacteria might be present in *JunB*$^{\Delta ep}$ skin (Figure S2).

(H) Representative images of *S. aureus* immunofluorescence staining of skin from control and *JunB*$^{\Delta ep}$ mice at 6–7 months ($n > 10$ per genotype). Arrow indicates *S. aureus* colonies.

(I) Percentage of *JunB*$^{\Delta ep}$ mice positive for *S. aureus* colonization, as assessed by IF for *S. aureus*.

(J) Outside-in barrier assay using toluidine blue dye at embryonic day 17.5 (n = 6, 6).

To determine the origin of *S. aureus* colonizing the skin of *JunB*$^{\Delta ep}$ mice, we carried out PCR analysis for genes that are human specific and rarely found in murine-adapted strains (Holtfreter et al., 2013). These include the immune invasion gene cluster including staphylokinase (*sak*) and staphylococcal complement inhibitor (*scn*) and a human-specific prophase integrase-gene, *sa3int*. In addition, we also analyzed the presence of *mecA*, the gene responsible for methicillin resistance (Figure S1C). Most of the colonies isolated from *JunB*$^{\Delta ep}$ skin were positive for *sak*, *scn*, and *sa3int* but were negative for *mecA*, indicating that these bacteria are most likely human adapted but not methicillin resistant. These data suggest that dysbiosis allows *S. aureus*, originating presumably from caretakers, to colonize the skin of *JunB*$^{\Delta ep}$ mice.

Barrier defects are a major hallmark of AD pathology and could explain the dysbiosis in *JunB*$^{\Delta ep}$ mice (De Benedetto et al., 2011; Weidinger et al., 2018). We therefore evaluated barrier function in *JunB*$^{\Delta ep}$ mice. Lesional skin from *JunB*$^{\Delta ep}$ mice exhibited significant trans-epidermal water loss, similar to AD (Figure S1D). Levels of loricrin, a component of the cornified cell envelope, were also lost in lesional areas in *JunB*$^{\Delta ep}$ mouse skin, consistent with compromised barrier function (Figure S1E). To investigate whether this was due to the absence of JunB or secondary to the skin inflammation, we examined *JunB*$^{\Delta ep}$ mouse skin prior to the onset of skin inflammation, specifically during embryonic development and neonatal life. We first evaluated *JunB*$^{\Delta ep}$ mice pre-birth, at embryonic day 17.5, when the skin barrier is already formed. Skin barrier function was evaluated using a well-established protocol (Indra and Leid, 2011) in which embryos were dipped into toluidine blue and assessed for skin penetrance. By this assay, we observed marked toluidine blue indicative of defective barrier function in *JunB*$^{\Delta ep}$ mice (Figure 1J). To assess barrier function post-birth, we measured trans-epidermal water loss. Surprisingly, at 1 and 7 days post-birth, the skin barrier appeared intact in the *JunB*$^{\Delta ep}$ mice, suggesting compensatory mechanisms (Figure S1D). Furthermore, we used the biotin diffusion assay (Schmitz et al., 2015), whereupon fluorescently labeled biotin was injected subcutaneously and the leakage of biotin past the tight junctions was evaluated 1 day post-birth. We observed that the injected biotin co-localized with occludin, staining the tight junctions, suggesting intact inside-out barrier function (Figure S1F). Occludin expression was more prominent in *JunB*$^{\Delta ep}$ mice compared with controls, consistent with a compensatory strengthening of tight junction formation. These data suggest that JunB is important for skin barrier function transiently before birth.

Overlapping Transcriptomic Signature with Human AD and Both Type 2 and Type 17 Immune Activation Are Observed in Lesional Skin of *JunB*$^{\Delta ep}$ Mice

Our results indicated that *JunB*$^{\Delta ep}$ mice exhibited clinical and histopathological similarities with cutaneous dysbiosis and AD. To determine whether the pathological features of *JunB*$^{\Delta ep}$ skin were transcriptionally similar to AD, we carried out RNA sequencing analysis from lesional skin of *JunB*$^{\Delta ep}$ mice at 6 months of age. Whole skin was isolated from three *JunB*$^{\Delta ep}$ mice and three controls. Total skin was lysed and mRNA extracted and subjected to RNA sequencing. We identified 615 differentially expressed genes (DEGs) with a false discovery rate (FDR) value of <0.05 between *JunB*$^{\Delta ep}$ and controls. These DEGs included genes involved in the immune/inflammatory response, cytokine signaling, keratinization, and regulation of defense responses, consistent with a chronic inflammatory skin phenotype with bacterial colonization, similar to what is observed in AD (Weidinger et al., 2018) (Figure 2A). Importantly, several pathways dysregulated in AD were also upregulated in our dataset, including IL-5, IL-23, IL-17, and Jak-Stat signaling pathways (Figure 2B), noteworthy because many of these pathways are presently under clinical investigation for the treatment of AD (Paller et al., 2017). We then carried out gene set enrichment analysis, comparing the *JunB*$^{\Delta ep}$ skin transcriptome with the MADAD (meta-analysis-derived AD) gene signature (Ewald et al., 2015), a meta-analysis-derived DEG list from human AD skin. We found a statistically significant positive correlation between the *JunB*$^{\Delta ep}$ skin and the MADAD transcriptome (Figure 2C, left).

To further investigate the immune responses in these mice, we compared the transcriptomic signature of lesional *JunB*$^{\Delta ep}$ skin with publicly available datasets investigating keratinocyte responses to two major pro-inflammatory cytokines present in AD, IL-4, and IL-17A. Strikingly, we observed a statistically significant positive correlation for both of these signatures to *JunB*$^{\Delta ep}$ skin, indicating that both type 2 and type 17 immune responses are active in *JunB*$^{\Delta ep}$ skin (Figure 2C, middle and right). A recent report comparing psoriasis and AD showed extensive overlap of these two diseases at the transcriptomic level (Tsoi et al., 2019). We evaluated whether expression of some of the genes selectively regulated in AD, such as *Duox1*, *Il13ra2*, *Stac2*, as well as other genes implicated in AD pathogenesis in mice or humans such as *Tslp*, *Aqp3*, *Il4ra*, *Nod2*, *Osm*, *Slc4a11*, *Mmp13*, and *Serpinb3* were altered in *JunB*$^{\Delta ep}$ mice (Olsson et al., 2006; Cianferoni and Spergel, 2014; Miyai et al., 2016; Weidinger et al., 2018). We observed an upregulation of a large proportion of these genes in *JunB*$^{\Delta ep}$ mice (Figure 2D). Moreover, we observed a statistically significant correlation of the transcriptome of *JunB*$^{\Delta ep}$ skin with that of ADAM17$^{\Delta ep}$ mice, recently published to model the AD transcriptome (Woodring et al., 2018) (Figure 2E).

Collectively, these data show that *JunB*$^{\Delta ep}$ mice have skin inflammation with hallmarks of AD both at the phenotypic and at the molecular level.

Skin Inflammation and *S. aureus* Colonization Are Exacerbated in the Absence of Adaptive Immunity

We then conducted a detailed analysis of the immune cells in the skin of *JunB*$^{\Delta ep}$ mice pre- and post-lesion development, which demonstrated involvement of both the innate and adaptive immune systems. Specifically, we observed increased monocytes, neutrophils, TCR$\alpha\beta$ T cells, and TCR$\gamma\delta$ T cells in *JunB*$^{\Delta ep}$ mice (Figures 3A–3C). Interestingly, TCR$\alpha\beta$ and TCR$\gamma\delta$ T cells were increased prior to epidermal hyperplasia, whereas neutrophils and monocytes were increased at later stages of disease (Figures 3A–3C). Furthermore, group 2 innate lymphoid cells (ILC2s), which produce IL-13 and have been proposed to contribute to the pathogenesis of AD (Kim et al., 2013; Roediger et al., 2013; Salimi et al., 2013), were also increased before

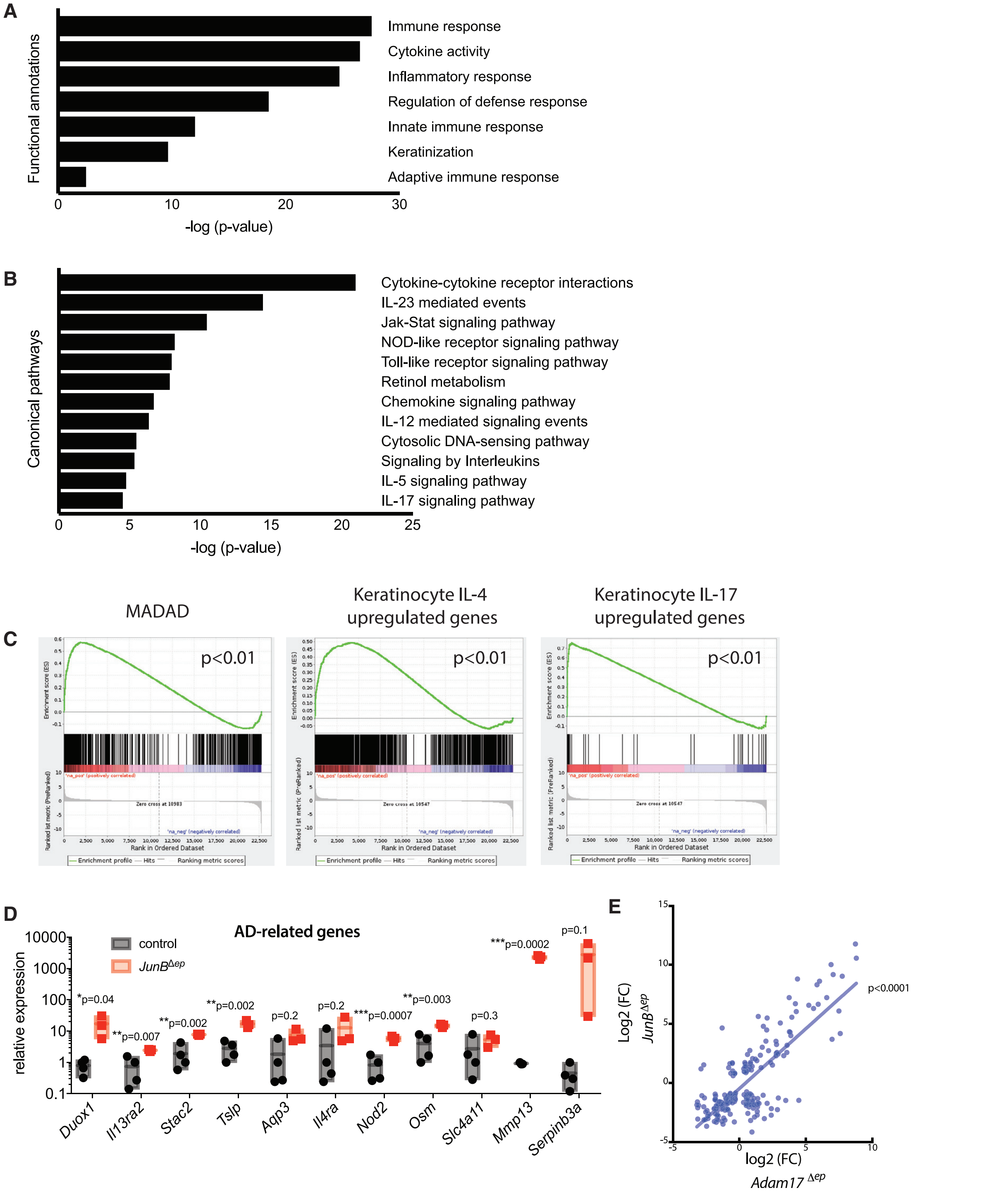

Figure 2. Lesional Skin from *JunB*$^{\Delta ep}$ Mice Shows Overlapping Transcriptomic Signature with Human AD, with Both Type 2 and Type 17 Immune Activation

(A) Functional annotations derived from RNA sequencing (RNA-seq) analysis of skin from control (n = 3) and *JunB*$^{\Delta ep}$ mice (n = 3).

(B) Canonical pathways derived from RNA-seq analysis of skin from control (n = 3) and *JunB*$^{\Delta ep}$ mice (n = 3).

(C) Gene set enrichment analysis (GSEA) showing positive correlations with MADAD (meta-analysis-derived AD) signature, IL-4- and IL-17-responsive genes in keratinocytes.

(D) Expression levels of genes implicated in AD pathology, as assessed by qPCR.

(E) Correlation of transcriptomic changes of commonly expressed genes in *JunB*$^{\Delta ep}$ mice versus *Adam17*$^{\Delta ep}$ mice (Woodring et al., 2018).

OPEN ACCESS
CellPress

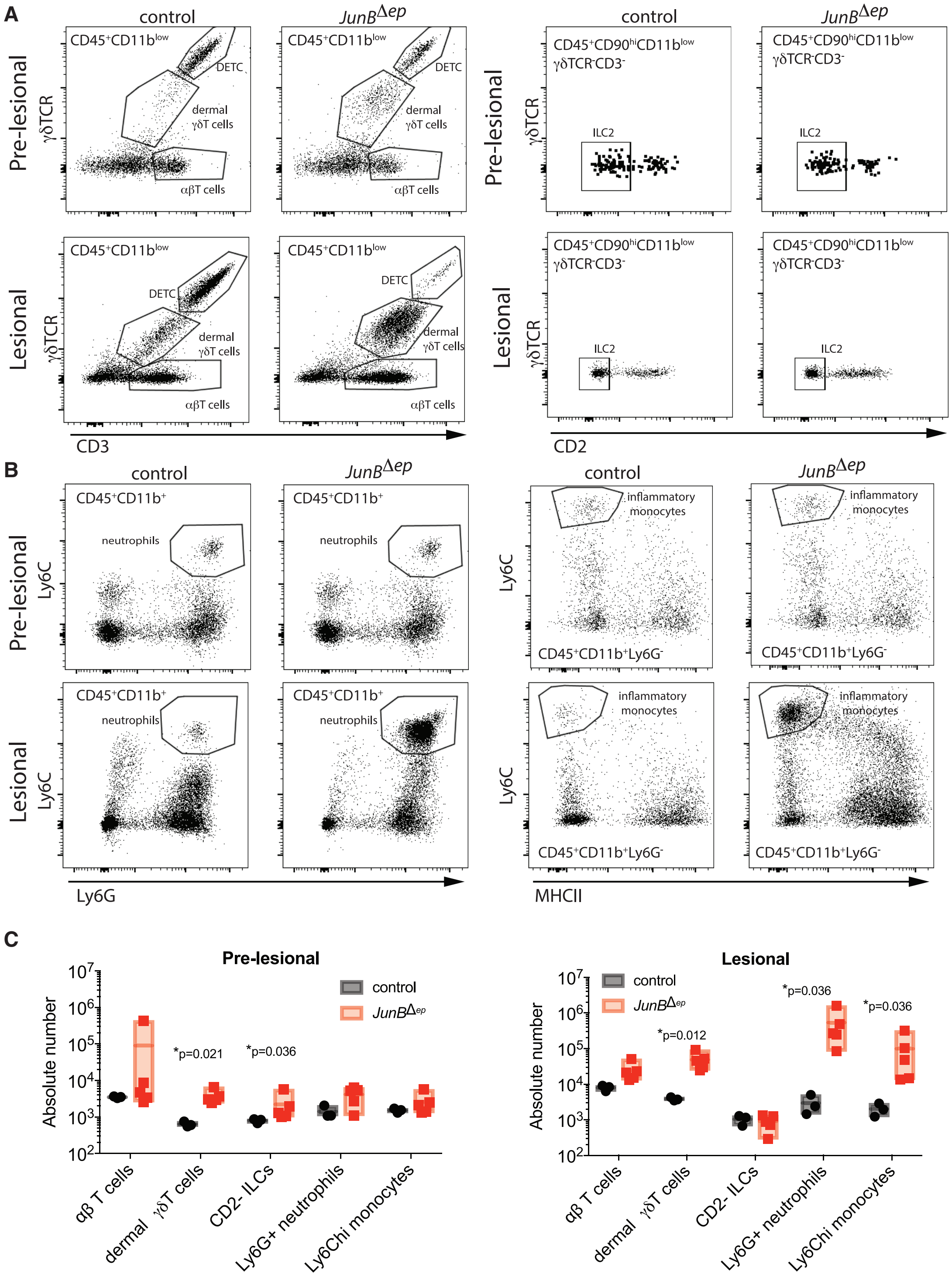

Figure 3. T Cells and ILC2s Infiltrate Skin before the Onset of Overt Inflammation

(A and B) Representative flow cytometry plots showing (A) T cell subsets, ILCs, and (B) neutrophils, inflammatory monocytes of pre-lesional (3 months of age; n = 3, 5) and lesional (6 months of age; n = 3, 5) skin in control and *JunB*$^{\Delta ep}$ mice.

(C) Absolute numbers of immune cell subsets as analyzed using flow cytometry of pre-lesional (3 months of age) and lesional (6 months of age) skin in control and *JunB*$^{\Delta ep}$ mice. Markers defining each cell subset are as follows: αβT cells: CD45^{+}CD11b$^{-/\,low}$CD3^{+}γδTCR^{-}; γδT cells: CD45^{+}CD11b$^{-/low}$CD3^{+}gdTCR^{int}; DETC: CD45^{+}CD11b$^{-/low}$CD3^{hi}gdTCR^{hi}; CD2− ILCs: CD45^{+}CD90^{hi}CD11b^{-}CD3^{-}γδTCR^{-}CD2^{-}; Ly6G+ neutrophils: CD45^{+}CD11b^{hi}CD3^{-}gdTCR^{-}Ly6G^{hi}; Ly6Chi monocytes: CD45^{+}CD11b^{+}CD3^{-}gdTCR^{-}Ly6G^{-}Ly6C^{hi}MHCII−.

epidermal hyperplasia, but their numbers normalized as inflammation worsened (Figure 3C).

The role of T cells in cutaneous dysbiosis and AD pathogenesis remains incompletely understood. Generally, it is considered that type 2 inflammatory cytokine production by T cells enables *S. aureus* colonization and that *S. aureus*-derived superantigens activate T cells in the skin to promote inflammation (Bieber, 2008). In contrast, IL-17 production by T cells is likely to serve anti-*S. aureus* function, partially via upregulation of anti-microbial proteins (Cho et al., 2010). To investigate whether T cells exacerbated or ameliorated inflammation in *JunB*$^{\Delta ep}$ mice with spontaneous dysbiosis, we crossed these mice to *Rag1*$^{-/-}$ mice, which lack mature T and B cells. Strikingly, *JunB*$^{\Delta ep}$*Rag1*$^{-/-}$ mice developed exacerbated skin inflammation at an earlier time point with higher *S. aureus* load (Figure 4A). In ~15% of *JunB*$^{\Delta ep}$*Rag1*$^{-/-}$ mice, *S. aureus* caused superinfections and botryomycosis (data not shown). We observed an increase in the number of mast cells by toluidine blue staining and Langerhans cells by langerin staining in *JunB*$^{\Delta ep}$*Rag1*$^{-/-}$ skin (Figure 4A). Importantly, we observed equal or higher amounts of pro-inflammatory cytokines in the 3-month-old *JunB*$^{\Delta ep}$*Rag1*$^{-/-}$ (lesional skin) mice compared with *JunB*$^{\Delta ep}$ mice (pre-lesional skin), including elevated IL-4, IL-1β, and IL-22, as well as alarmins and the anti-microbial proteins beta-defensin 14, Lipocalin-2 (Lcn-2), and S100A9 (Figure 4B), confirming that the inflammatory mediators appear earlier in *JunB*$^{\Delta ep}$*Rag1*$^{-/-}$ compared with *JunB*$^{\Delta ep}$ *mice*.

These data suggest that T cells are important for controlling *S. aureus* in *JunB*$^{\Delta ep}$ mice, presumably via IL-17A production, even though we cannot exclude the role of B cells, also absent in *Rag1*$^{-/-}$ mice. Consistent with this hypothesis, *JunB*$^{\Delta ep}$ mice had higher serum IL-17A levels compared with *JunB*$^{\Delta ep}$*Rag1*$^{-/-}$ mice (Figure 4C). IL-17A can be produced by both TCRαβ T cells and TCRγδ T cells, as well as ILC3s (Sumaria et al., 2011; Spits et al., 2013; Ebbo et al., 2017). To determine the cell type(s) responsible for IL-17A production in *JunB*$^{\Delta ep}$ mice, we crossed these mice to an *Il17a*-eGFP reporter mouse (Lee et al., 2012). In these mice, cells expressing *Il17a* express GFP, which is readily detectable using flow cytometry. We observed basal *Il17a*-eGFP expression by skin-resident TCRγδ T cells but not TCRαβ T cells in control mice and a massive upregulation of *Il17a* expression by both TCRγδ and TCRαβ T cells (Th17 cells) in *JunB*$^{\Delta ep}$ skin (Figure 4D).

IL-17 can also be produced by ILC3s (Ebbo et al., 2017). Cytokine production by ILC3s has been shown to be capable of compensating for the absence of T cells to prevent colonic infection with *Citrobacter rodentium* (Rankin et al., 2016). We observed only modest *Il17a* expression in skin ILCs in (Rag-sufficient) *JunB*$^{\Delta ep}$ skin (Figure 4E). However, we were interested in determining whether this changed in the absence of T cells. We therefore examined IL-17A expression in *JunB*$^{\Delta ep}$*Rag1*$^{-/-}$ mice on a *Il17a*-eGFP reporter background. In these mice, we observed *Il17a*-eGFP expression only in ILCs, presumably ILC3s. However, these *Il17a*$^{+}$ ILC3s were substantially less abundant than *Il17a*$^{+}$ T cells in (Rag-sufficient) *JunB*$^{\Delta ep}$ skin (Figure 4C). This was confirmed in unbiased t-Distributed Stochastic Neighbor Embedding (t-SNE) analysis of the *Il17a*-eGFP^{+} cells in *JunB*$^{\Delta ep}$ and *JunB*$^{\Delta ep}$*Rag1*$^{-/-}$ mice, which indicated an increase in the number of GFP^{+} ILC3s in *JunB*$^{\Delta ep}$*Rag1*$^{-/-}$ mice relative to *JunB*$^{\Delta ep}$ mice that was nevertheless substantially short of the T cell response observed in Rag-sufficient mice (Figure 4E).

To determine whether ILC3s can compensate for T cells in a setting where IL-17A is potently induced, we topically administered imiquimod (IMQ) to Rag-sufficient or *Rag1*$^{-/-}$ mice for 5 days. We observed *Il17a*-eGFP expression in Th17, TCRγδ, and ILCs upon IMQ administration to Rag-sufficient mice (Figures S3A and S3B). However, in *Rag1*$^{-/-}$ mice, the *Il17a*-eGFP expression in ILC3s was not increased. Thus, this limited capacity for *Il17a*$^{+}$ ILC3s to compensate for T cells was observed in both settings (i.e., *JunB*$^{\Delta ep}$*Rag1*$^{-/-}$ and following topical treatment with IMQ, a potent stimulator of IL-17) (Figure S3C).

In summary, in the absence of the adaptive immune system, *S. aureus* colonization and skin inflammation were accelerated and exacerbated, and this correlated with diminished IL-17A production within the skin.

Prophylactic Antibiotics Reduce Skin Inflammation before Microbial Dysbiosis

Antibiotics are often used in AD patients with limited efficacy, hypothesized to be due to lack of specificity to *S. aureus* and possibly lack of effect on *S. aureus* killing (Williams et al., 2017). Indeed, understanding of the role of microbial dysbiosis in AD is confounded by variable response to antibiotic treatment. Thus, whether *S. aureus* colonization is causal or a consequence of type 2 inflammation remains to be answered. To investigate the pathological role of *S. aureus* colonization in the skin inflammation observed in *JunB*$^{\Delta ep}$ mice, we treated these mice with antibiotics pre- and post-*S. aureus* colonization. We used the *JunB*$^{\Delta ep}$ mice crossed to *Il17a*-GFP reporter mice to analyze IL-17A responses. Mice were put on antibiotics in their drinking water at weaning and observed until their non-antibiotic-treated counterparts developed skin inflammation (6–7 months). Importantly, at 1 month of age, no colonization of *S. aureus* was observed, even though some colonies of Gram-negative bacteria were observed in *JunB*$^{\Delta ep}$ skin (data not shown). We observed a strong amelioration of the skin inflammatory phenotype in *JunB*$^{\Delta ep}$ mice treated with antibiotics, with reduced hyperkeratosis and decreased immune cell infiltration (Figure 5A). Gram staining identified decreased to non-detectable Gram-positive colonization on the skin of mice treated with antibiotics (Figure 5A). Swabs taken from the skin of mice and grown on blood agar plates showed reduced *S. aureus* burden in mice treated with antibiotics (Figure 5B). Detailed flow cytometry analysis of the skin of these mice demonstrated decreased IL-17A-producing γδT cells as well as Th17 cells and ILCs (Figures 5C–5E). We also observed a drop in the number of neutrophils and inflammatory macrophages in the skin of *JunB*$^{\Delta ep}$ mice treated with antibiotics before the development of skin inflammation and *S. aureus* colonization. These data suggest that *S. aureus* colonization contributes to the development of chronic skin inflammation in *JunB*$^{\Delta ep}$ mice.

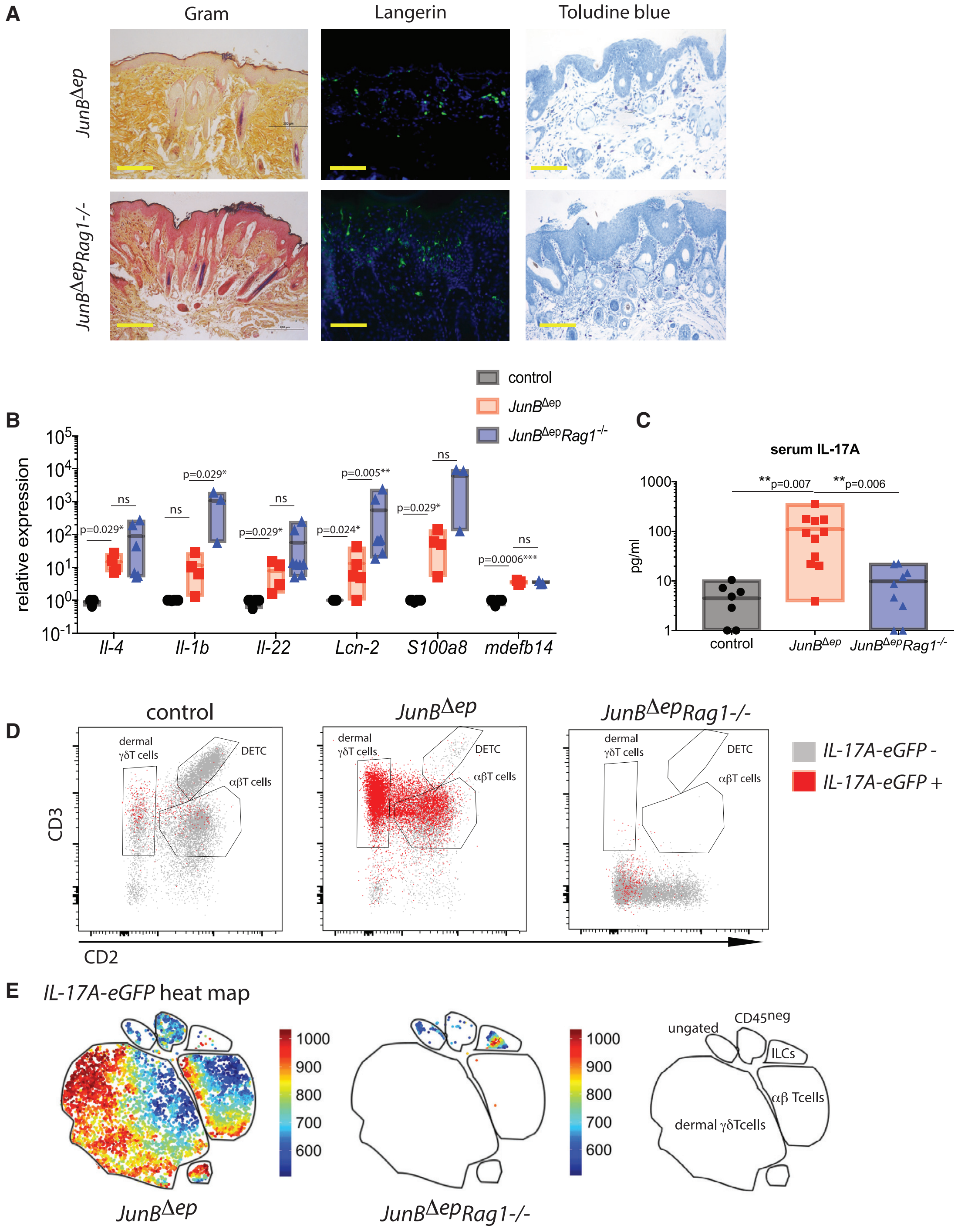

Figure 4. Skin Inflammation and *S. aureus* Colonization Are Exacerbated in the Absence of Adaptive Immunity

(A) Representative images of Gram, langerin, and toluidine blue of skin in $JunB^{\Delta ep}$ and $JunB^{\Delta ep}Rag1^{-/-}$ mice at 3 months of age (n = 6, 6). The yellow scale bar indicates 200 μm.

(B) Relative expression levels of pro-inflammatory cytokines in the skin of $JunB^{\Delta ep}$ and $JunB^{\Delta ep}Rag1^{-/-}$ mice at 3 months of age compared with controls (n = 3, 5, 6).

(C) Serum IL-17A levels in $JunB^{\Delta ep}$ and $JunB^{\Delta ep}Rag1^{-/-}$ mice at 3 months of age compared with controls (n = 7, 11, 9).

(D) Representative flow cytometry plots with GFP+ cells overlaid in the skin of $JunB^{\Delta ep}$ and $JunB^{\Delta ep}Rag1^{-/-}$ mice at 4 months of age compared with controls. The cells shown are gated on $CD45^{+}CD90^{+}CD11b^{-}$.

(E) Heatmaps showing IL-17A expression on t-SNE plots from total IL-17A-eGFP+ cells. The map on the right is created using conventional gating, as described in Figure 2.

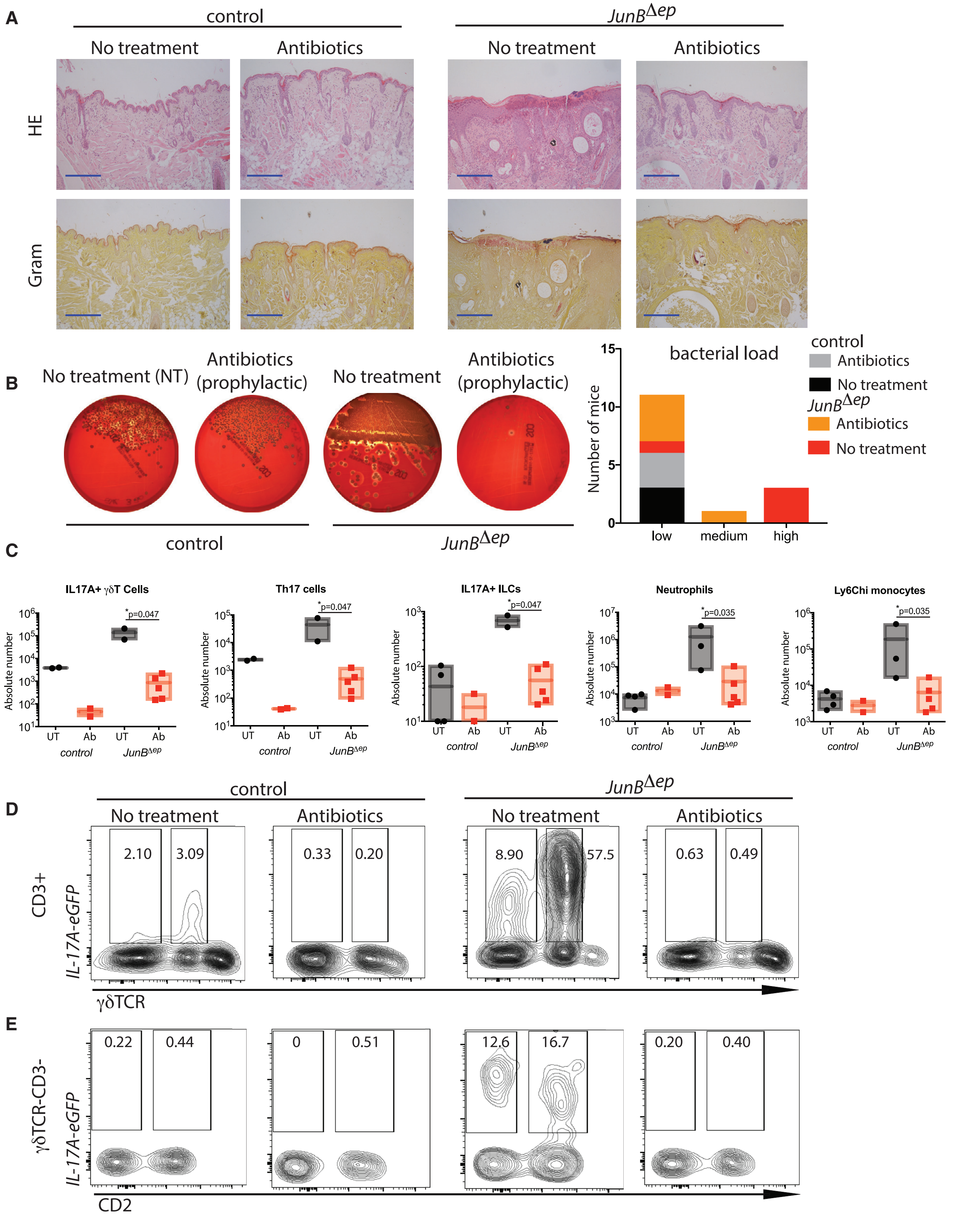

(legend on next page)

Antibiotic Treatment Post-development of Skin Inflammation Does Not Provide Therapeutic Benefit

Because we observed decreased skin inflammation when mice were treated with antibiotics before skin inflammation and dysbiosis occurred, we then moved to a therapeutic model. *JunB*$^{\Delta ep}$ *Il17a*-GFP reporter mice were put on antibiotics in their drinking water after the development of skin inflammation at 6 months for either 1 or 2 weeks, prior to immunological assessment as above.

t-SNE analysis, as well as conventional flow cytometry analysis, of the lymphoid and the myeloid cells in the skin of mice upon either 1 or 2 weeks of antibiotic treatment illustrated a transient decrease in the total number of αβ and γδT cells, with no major changes in neutrophil and monocyte numbers (Figure 6A; Figures S4 and S5). With regard to the IL-17A-producing cells, at 1 week, the effect of antibiotics was minimal, with a slight trend towards reduced numbers of *Il17a*-GFP^{+} γδT cells and ILC3s in skin of mice treated with antibiotics (Figures 6A and 6B). At 2 weeks post-antibiotics, we observed a statistically significant decrease in the number of *Il17a*-GFP^{+} γδT cells (Figures 6A and 6B; Figure S4). Unexpectedly, the number of skin neutrophils was increased 1 week post-antibiotics, but this trend did not continue 2 weeks post-antibiotics (Figure 6B; Figure S4). αβT cell numbers were increased in the skin-draining lymph nodes of these mice, whereas no changes were observed in the number *Il17a*-GFP^{+} γδT cells, Th17 cells, neutrophils, or monocytes (Figure S6). Swabs from mice grown on blood agar plates showed a general reduction of *S. aureus* colonies upon antibiotic treatment, although the extent of reduction was variable (Figure 6C). To determine whether antibiotic resistance might account for the variability observed in the different mice, we subjected another set of *JunB*$^{\Delta ep}$ mice at 6 months of age to antibiotics for 3 weeks, following the bacterial burden over time. Interestingly, not all mice behaved the same upon antibiotic treatment. Although some mice were sensitive to antibiotics, others were completely resistant (Figure S7A). Flow cytometric analysis at the 3 week time point showed that mice that were sensitive to antibiotics had smaller numbers of γδT cells, αβT cells, ILCs, neutrophils, and inflammatory monocytes in the skin compared with resistant mice (Figure S7B). Of note, these short antibiotic treatment schemes did not lead to any discernable macroscopic changes in the clinical features of these mice (data not shown). These data suggest that IL-17A-producing cells are linked to the bacterial burden, and antibiotic treatment post-dysbiosis leads to a transient induction in macrophage and neutrophil numbers. However, resistance occurs in a subset of mice upon continued antibiotic administration. Additional studies will be needed to delineate the differences in the microbiota composition of the mice showing antibiotic sensitivity versus resistance.

MyD88 Signaling Is Upregulated in *JunB*$^{\Delta ep}$ Skin, and MyD88 Is Upregulated in JunB-Deficient Keratinocytes

IL-36α has recently been implicated to be upregulated by *S. aureus* and orchestrate the downstream IL-17 signaling events (Liu et al., 2017; Nakagawa et al., 2017). Consistent with this, we observed elevated *Il36a* and *Il36b* and *Il36g* expression levels in lesional skin of *JunB*$^{\Delta ep}$ mice (Figure 7A). To determine whether the trigger for the elevated *Il36a* levels was due to microbial signals or deletion of *JunB*, we turned to *in vitro* keratinocyte cultures. Specifically, keratinocytes isolated from *JunB*$^{lox/lox}$ mice were subjected to infection by adenoviruses expressing Cre recombinase and empty adenoviruses as control. *JunB*-deficient and *JunB*-sufficient keratinocytes were then exposed to microbial antigens produced from supernatants derived from swabs applied onto *JunB*$^{\Delta ep}$ lesional skin (Figure 7B). Deletion of *JunB* in keratinocytes *in vitro* led to higher *Il36a* relative to controls, which was not further enhanced by microbial signals (Figure 7C). Expression of genes encoding several alarmins, such as the S100A9 and Lcn-2, as well as inflammation-associated chemokines Cxcl1, Cxcl2, and Ccl2 were upregulated in keratinocytes upon incubation with the microbial supernatant, and these responses were further upregulated in keratinocytes lacking JunB (Figure 7C).

As MyD88 is downstream of IL-36 signaling, we checked whether MyD88-mediated signaling is upregulated in *JunB*$^{\Delta ep}$ lesional skin. MyD88 was predicted as one of the upstream regulators of the global changes in gene expression by Ingenuity Pathway Analysis (IPA), and many genes downstream of MyD88 were significantly regulated (Figure 7D). Moreover, an increase in MyD88 protein levels was observed in keratinocytes lacking JunB, which was not further increased upon microbial signals (Figure 7E). *In vivo*, we observed increased levels of MyD88 protein in keratinocytes and immune cells in *JunB*$^{\Delta ep}$ skin, as assessed by immunohistochemistry (Figure 7F). These data suggest that JunB negatively regulates IL-36 and MyD88 levels in keratinocytes, which have been shown to be important for the colonization of *S. aureus* on skin (Figure 7G).

DISCUSSION

In this study, we show that epithelial expression of JunB is a gatekeeper of immune-microbiota interactions, and its loss leads to chronic skin inflammation with spontaneous *S. aureus* colonization. *JunB*$^{\Delta ep}$ mice share many pathological aspects of AD, including but not limited to type 2 and type 17 immune responses, elevated IgE levels, barrier dysfunction, and, importantly, spontaneous *S. aureus* colonization.

Figure 5. Prophylactic Antibiotics Reduce Skin Inflammation

(A) Representative H&E and Gram staining images of control and *JunB*$^{\Delta ep}$ mice with or without prophylactic antibiotics (UT: n = 4, 3; Ab: n = 2, 5). The blue scale bar indicates 200 μm.

(B) Blood agar plates from swabs from control and *JunB*$^{\Delta ep}$ skin with or without prophylactic antibiotic treatment and quantification of total bacterial load on skin.

(C) Number of infiltrating immune cells into the skin of control and *JunB*$^{\Delta ep}$ mice with or without prophylactic antibiotics as analyzed using flow cytometry. Gating strategy as described in Figure 2.

(D) Representative flow cytometry images of IL-17A expressing T cells of control and *JunB*$^{\Delta ep}$ mice with or without prophylactic antibiotics.

(E) Representative flow cytometry images of IL-17A expressing ILCs of control and *JunB*$^{\Delta ep}$ mice with or without prophylactic antibiotics.

OPEN ACCESS
CellPress

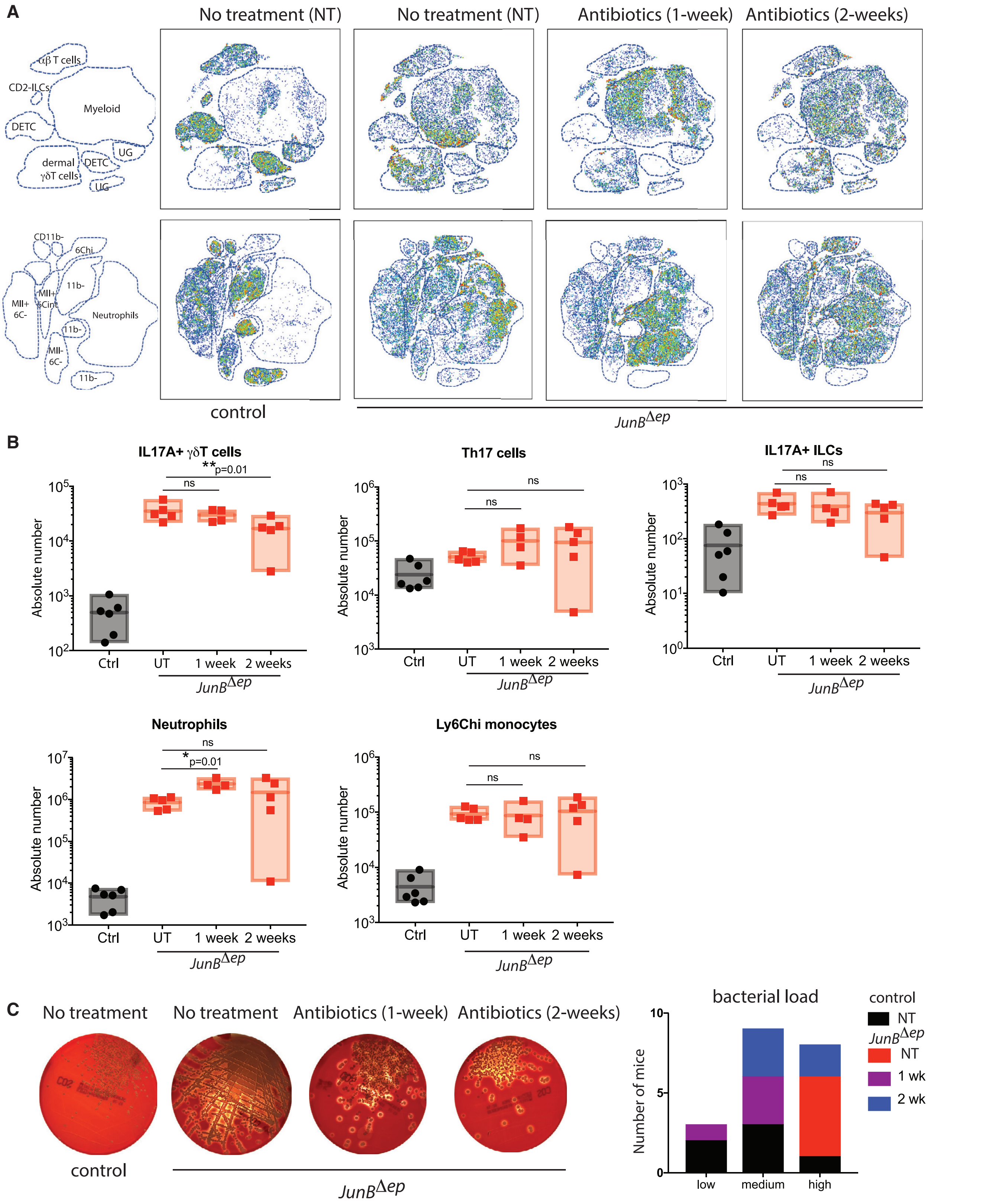

(legend on next page)

We took advantage of this mouse model to address some unanswered questions, such as the role of T cells in controlling *S. aureus* and the role of IL-17A in AD with *S. aureus* colonization.

IL-17, a potent pro-inflammatory cytokine, is central to the pathogenesis of psoriasis, and IL-17 blockers have shown unprecedented success in the treatment of this disease (Lebwohl et al., 2011; Langley et al., 2014; Ratner, 2015). IL-17 is produced by T helper cell subset Th17, as well as $\gamma\delta$T cells and ILC3s. IL-17 is upregulated in AD skin, albeit at lower levels compared with psoriasis, but its role in AD remains unclear (Guttman-Yassky et al., 2008). Whether IL-17A has a protective or pathogenic role in pediatric or adult AD remains to be understood. Elucidating the role of IL-17A in AD is important, as blockade of IL-17A and its upstream cytokine IL-23 is currently being tested in clinical trials for AD (Paller et al., 2017).

ILCs have been proposed to play a protective role in *S. aureus*- and pneumonia-induced inflammatory processes (Rak et al., 2016; Gray et al., 2017), and ILC2s have been shown to accumulate in the skin of AD patients (Kim et al., 2013; Salimi et al., 2013). Kinetic analysis of the ILCs in the skin of *JunB*$^{\Delta ep}$ mice showed a transient increase in the number of ILCs before the appearance of overt skin inflammation and *S. aureus* colonization. However, there were no differences in the number of ILCs infiltrating the lesional skin, whereas an increased number of IL-17A+ ILCs (ILC3s) were observed. In the presence of T cells, we observed a minor contribution of ILC3s to the IL-17A-producing population. However, in *Rag1*$^{-/-}$ mice, the number of IL-17A expressing ILC3s increased, albeit not to sufficient levels to compensate for the T cell deficit. These data suggest that ILC3s are only partially redundant with T cells in the skin. Future studies are needed to determine whether ILC3s are present in AD skin and the level of plasticity observed between group 2 and group 3 ILCs in AD.

Barrier defects, caused for example by filaggrin mutations, have been linked to the pathogenesis of AD. However, it is important to note that about 40% of individuals with filaggrin mutations never show AD symptoms (ORegan et al., 2009). Thus, it is likely that barrier defects are not sufficient to lead to the development of AD, and a second hit, possibly from the environment, is needed to induce the inflammatory responses in skin. Along with these findings in humans, the filaggrin-deficient mice, which have a clear skin barrier defect, do not develop inflammation until 20–28 weeks of age (Oyoshi et al., 2009). Moreover, filaggrin defects are shown not to be sufficient to induce spontaneous AD-like lesions in many reports. Most studies add an allergen such as house dust mite to induce inflammation in mice with filaggrin defects (Moniaga and Kabashima, 2011; Kawasaki et al., 2012). We hypothesize that in the *JunB*$^{\Delta ep}$ mice, the early-life barrier dysfunction renders the skin more sensitive to allergens and microbes, in this case, particularly *S. aureus*.

The dysbiosis observed in *JunB*$^{\Delta ep}$ mice likely precedes inflammation, as prophylactic antibiotic treatment restricted the development of skin inflammation and strongly ameliorated epidermal hyperproliferation. In contrast, antibiotic treatment after *S. aureus* colonization demonstrated variable efficacy in *JunB*$^{\Delta ep}$ mice. This mirrors the clinical reality of AD, in which antibiotic treatment often demonstrates little to no success in controlling disease. The reason for the disparate efficacy of prophylactic versus therapeutic antibiotic treatment remains unknown. This may reflect antibiotic access or efficacy within inflamed skin and/or active drug resistance mechanisms in established *S. aureus*. Alternatively, there may be other species of antibiotic-susceptible bacteria that are necessary for *S. aureus* colonization but dispensable once colonization has established. In *JunB*$^{\Delta ep}$ mice, we detected small amounts of Gram-negative bacteria at early time points, before the colonization of *S. aureus*, that did not correlate with disease. Thus, although we cannot formally exclude that other bacteria might initiate dysbiosis in these mice, because of their low abundance and variability, they are unlikely to be directly responsible for the observed pathology. With growing advances in microbiome sequencing technology, these questions will be addressable in the near future.

The mechanisms by which *S. aureus* is sensed by different cell types in the skin and the downstream signaling pathways activated have recently gained a lot of attention (Bitschar et al., 2017). Pattern recognition receptors such as Toll-like receptors (TLRs), as well as NOD receptors, have been shown to contribute to the initial recognition of bacterial antigens. Activation of the TLRs leads to NF-κB activation via MyD88 signaling (Kawai and Akira, 2010). Mutations in TLR2 decreasing intracellular signaling have been linked to higher *S. aureus* load and disease severity in AD (Bin and Leung, 2016). However, the mechanisms leading to susceptibility to develop AD are still not well understood. An important finding of our study is the observation that JunB loss in keratinocytes is sufficient to drive skin inflammation, likely through a combination of dysbiosis due to barrier dysfunction and the enhanced responses to dysbiosis. Mechanistically, JunB negatively regulates MyD88, because its deletion in keratinocytes *in vitro* is sufficient for upregulation of many chemokines and alarmins, including IL-36α, previously reported to be secreted from keratinocytes upon *S. aureus* colonization (Liu et al., 2017). The transcriptomic profile of the *JunB*$^{\Delta ep}$ skin shows regulation of several pathways such as TLR signaling, as well as many anti-microbial molecules, such as Lcn-2. Interestingly, deletion of p65 in keratinocytes in *JunB*$^{\Delta ep}$ mice led to exacerbated skin inflammation with higher *S. aureus* load,

Figure 6. Antibiotic Treatment Post-development of Skin Inflammation Does Not Provide Therapeutic Benefit

(A) t-SNE plots showing the distribution of lymphoid (top) and myeloid (bottom) cells in *JunB*$^{\Delta ep}$ mice with or without 1 and 2 weeks of antibiotics compared with controls as analyzed using flow cytometry. Maps on the left were made using conventional gating as described in Figure 2. UG, ungated (control [Ctrl], n = 6; *JunB*$^{\Delta ep}$ UT, n = 5; 1 week, n = 4; 2 weeks, n = 5).

(B) Number of infiltrating immune cells into the skin of *JunB*$^{\Delta ep}$ mice with or without 1 and 2 weeks of antibiotics compared with controls as analyzed by conventional flow cytometry.

(C) Blood agar plates from swabs from *JunB*$^{\Delta ep}$ mice with or without 1 and 2 weeks of antibiotics compared with controls as analyzed using flow cytometry and quantification of total bacterial load on lesional skin.

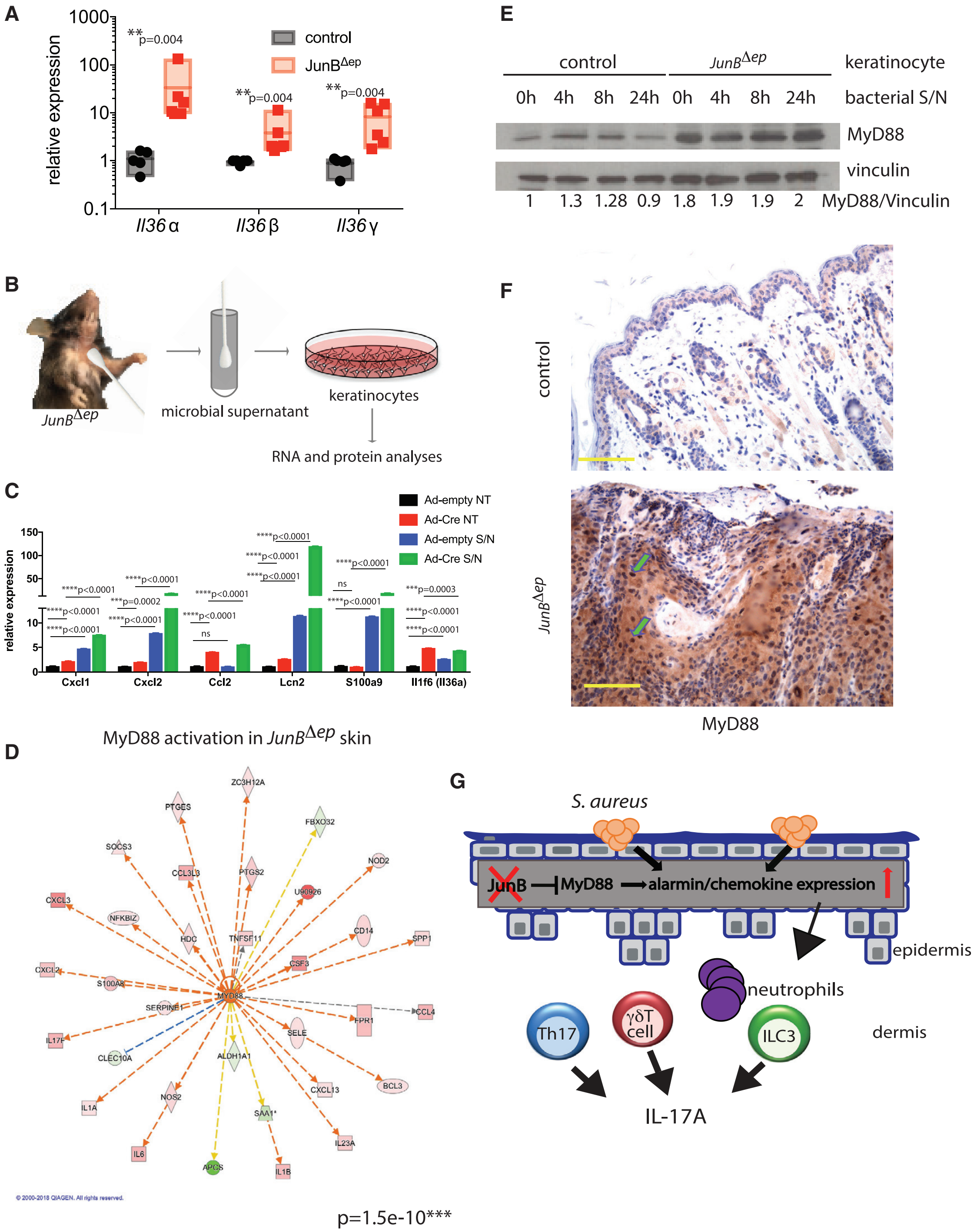

Figure 7. MyD88 Signaling Is Upregulated in *JunB$^{\Delta ep}$* Skin, and MyD88 Is Upregulated in JunB-Deficient Keratinocytes

(A) Relative expression levels of il1f6 (Il36α), Il1f8 (Il36β), and Il1f9 (Il36γ) in the lesional skin of *JunB$^{\Delta ep}$* mice compared with controls (n = 5, 6).

(B) Schematic diagram depicting the experimental design for (C) and (E).

(C) Relative expression levels of chemokines and alarmins upon JunB deletion in *JunB$^{lox/lox}$* keratinocytes using adenoviruses expressing Cre recombinase with or without supernatant (S/N) derived from swabs from lesional skin of *JunB$^{\Delta ep}$* mice (n = 3, 3, repeated three separate times).

(D) MyD88 and its downstream genes are significantly regulated in the transcriptome of *JunB$^{\Delta ep}$* skin.

(E) Western blot using control or *JunB$^{\Delta ep}$* keratinocytes upon S/N administration (as described in B) for indicated time points. MyD88 levels and vinculin (as loading control) are shown.

(legend continued on next page)

highlighting that the NF-κB pathway in keratinocytes is protective for skin homeostasis in the presence of *S. aureus* (data not shown). These data suggest that loss of JunB in keratinocytes is a susceptibility factor for the development of *S. aureus*-induced skin inflammation. Unfortunately, there are no protein data available from patients with overt *S. aureus* colonization and superinfections compared with non-overt/limited *S. aureus* colonization to determine the co-localization of bacterial colonies and keratinocyte JunB expression. A prospective study making use of biopsies without prior desterilization conditions will be necessary to answer this question.

In summary, we show that JunB is a critical transcription factor in immune-microbiota crosstalk and that its loss is detrimental for skin immunity. These findings highlight the essential role of keratinocytes, an underestimated cell type in skin inflammation. We also show that JunB negatively regulates MyD88 in keratinocytes. These mice are an ideal model to further investigate the role of the microbiota and *S. aureus* in AD, as they represent the natural evolution of skin inflammation resembling AD.

STAR★METHODS

Detailed methods are provided in the online version of this paper and include the following:

- KEY RESOURCES TABLE
- LEAD CONTACT AND MATERIALS AVAILABILITY
- EXPERIMENTAL MODEL AND SUBJECT DETAILS
  - Mice
- METHOD DETAILS
  - Histological analyses
  - Barrier analysis
  - Flow cytometry
  - Antibiotic administration
  - Bacterial analysis
  - Transcriptomic analysis
- QUANTIFICATION AND STATISTICAL ANALYSIS
- DATA AND CODE AVAILABILITY

SUPPLEMENTAL INFORMATION

Supplemental Information can be found online at https://doi.org/10.1016/j.celrep.2019.09.042.

ACKNOWLEDGMENTS

We thank the Wagner lab for helpful suggestions and discussion throughout the evolution of this project, specifically Alvaro Ucero, Nuria Gago, and Liliana Mellor. We thank Vanessa Bermeo and Guillermo Medrano for help with animal husbandry and genotyping. Ö.U. was funded by the ECTS/Amgen Bone Biology Fellowship (2013–2016) and by the Spanish Ministry of Economy and Competitiveness (SAF2012-39670). B.R. and W.W. were funded by a Jesus-Serra visiting scientist grant. E.F.W. was funded by a European Research Council advanced grant (ERC FCK/2008/37).

AUTHOR CONTRIBUTIONS

Ö.U. designed and performed the experiments and wrote the manuscript. M.J. contributed to experimental design and performed experiments. B.R. contributed to experimental design, data interpretation, and writing the manuscript. J.S. and L.T.D.-C. performed experiments. K.T. carried out the RNA sequencing analysis. W.W. contributed to the interpretation of experiments and writing of the manuscript. E.F.W. contributed to data interpretation, oversaw the project, and contributed to writing the manuscript.

DECLARATION OF INTERESTS

Ö.U. and B.R. are presently employees of the Novartis Institutes for Biomedical Research. Novartis did not fund the study, nor did it play any role in the study design, data collection and analysis, decision to publish, or preparation of the manuscript. All other authors declare no competing interests.

Received: January 23, 2019
Revised: July 1, 2019
Accepted: September 13, 2019
Published: October 22, 2019

REFERENCES

Bieber, T. (2008). Atopic dermatitis. N. Engl J. Med. *358*, 1483–1494.

Bin, L., and Leung, D.Y.M. (2016). Genetic and epigenetic studies of atopic dermatitis. Allergy Asthma Clin. Immunol. *12*, 52.

Bird, L. (2005). Asthma and allergy: TSLP: key role in allergic responses confirmed. Nat. Rev. Immunol. *5*, 747.

Bitschar, K., Wolz, C., Krismer, B., Peschel, A., and Schittek, B. (2017). Keratinocytes as sensors and central players in the immune defense against Staphylococcus aureus in the skin. J. Dermatolog. Sci. *87*, 215–220.

Brunner, P.M., Israel, A., Zhang, N., Leonard, A., Wen, H.C., Huynh, T., Tran, G., Lyon, S., Rodriguez, G., Immaneni, S., et al. (2018). Early-onset pediatric atopic dermatitis is characterized by TH2/TH17/TH22-centered inflammation and lipid alterations. J. Allergy Clin. Immunol. *141*, 2094–2106.

Cevikbas, F., and Steinhoff, M. (2012). IL-33: a novel danger signal system in atopic dermatitis. J. Invest. Dermatol. *132*, 1326–1329.

Chen, Y.E., Fischbach, M.A., and Belkaid, Y. (2018). Skin microbiota-host interactions. Nature *553*, 427–436.

Cho, J.S., Pietras, E.M., Garcia, N.C., Ramos, R.I., Farzam, D.M., Monroe, H.R., Magorien, J.E., Blauvelt, A., Kolls, J.K., Cheung, A.L., et al. (2010). IL-17 is essential for host defense against cutaneous Staphylococcus aureus infection in mice. J. Clin. Invest. *120*, 1762–1773.

Cianferoni, A., and Spergel, J. (2014). The importance of TSLP in allergic disease and its role as a potential therapeutic target. Expert Rev. Clin. Immunol. *10*, 1463–1474.

De Benedetto, A., Rafaels, N.M., McGirt, L.Y., Ivanov, A.I., Georas, S.N., Cheadle, C., Berger, A.E., Zhang, K., Vidyasagar, S., Yoshida, T., et al. (2011). Tight junction defects in patients with atopic dermatitis. J. Allergy Clin. Immunol. *127*, 773–786.e7.

Doherty, T.A., Khorram, N., Lund, S., Mehta, A.K., Croft, M., and Broide, D.H. (2013). Lung type 2 innate lymphoid cells express cysteinyl leukotriene receptor 1, which regulates TH2 cytokine production. J. Allergy Clin. Immunol. *132*, 205–213.

Ebbo, M., Crinier, A., Vély, F., and Vivier, E. (2017). Innate lymphoid cells: major players in inflammatory diseases. Nat. Rev. Immunol. *17*, 665–678.

(F) Representative images of MyD88 levels by immunohistochemistry in skin from control or *JunB*$^{\Delta ep}$ mice. Arrows indicate examples of MyD88-positive keratinocytes. The yellow scale bar indicates 100 μm.
(G) Upon loss of keratinocyte expression of JunB, MyD88 levels are increased. Together with microbial products, increased MyD88 levels lead to increased alarmin/chemokine expression, starting a cascade of inflammatory response, including increased IL-17A production from Th17, γδT cells, and ILC3s. In a *Rag1*$^{-/-}$ background, IL-17A is expressed by ILC3s, but skin inflammation is exacerbated in the absence of the adaptive immune system.

Ewald, D.A., Malajian, D., Krueger, J.G., Workman, C.T., Wang, T., Tian, S., Litman, T., Guttman-Yassky, E., and Suárez-Fariñas, M. (2015). Meta-analysis derived atopic dermatitis (MADAD) transcriptome defines a robust AD signature highlighting the involvement of atherosclerosis and lipid metabolism pathways. BMC Med. Genomics *8*, 60.

Ewald, D.A., Noda, S., Oliva, M., Litman, T., Nakajima, S., Li, X., Xu, H., Workman, C.T., Scheipers, P., Svitacheva, N., et al. (2017). Major differences between human atopic dermatitis and murine models, as determined by using global transcriptomic profiling. J. Allergy Clin. Immunol. *139*, 562–571.

Floudas, A., Saunders, S.P., Moran, T., Schwartz, C., Hams, E., Fitzgerald, D.C., Johnston, J.A., Ogg, G.S., McKenzie, A.N., Walsh, P.T., and Fallon, P.G. (2017). IL-17 receptor A maintains and protects the skin barrier to prevent allergic skin inflammation. J. Immunol. *199*, 707–717.

Geoghegan, J.A., Irvine, A.D., and Foster, T.J. (2018). Staphylococcus aureus and atopic dermatitis: a complex and evolving relationship. Trends Microbiol. *26*, 484–497.

Gong, J.Q., Lin, L., Lin, T., Hao, F., Zeng, F.Q., Bi, Z.G., Yi, D., and Zhao, B. (2006). Skin colonization by Staphylococcus aureus in patients with eczema and atopic dermatitis and relevant combined topical therapy: a double-blind multicentre randomized controlled trial. Br. J. Dermatol. *155*, 680–687.

Graña, O., Rubio-Camarillo, M., Fdez-Riverola, F., Pisano, D.G., and Glez-Peña, D. (2018). Nextpresso: next generation sequencing expression analysis pipeline. Curr. Bioinform. *13*, 583–591.

Gray, J., Oehrle, K., Worthen, G., Alenghat, T., Whitsett, J., and Deshmukh, H. (2017). Intestinal commensal bacteria mediate lung mucosal immunity and promote resistance of newborn mice to infection. Sci. Transl. Med. *9*, eaaf9412.

Guinea-Viniegra, J., Jiménez, M., Schonthaler, H.B., Navarro, R., Delgado, Y., Concha-Garzón, M.J., Tschachler, E., Obad, S., Daudén, E., and Wagner, E.F. (2014). Targeting miR-21 to treat psoriasis. Sci. Transl. Med. *6*, 225re1.

Guttman-Yassky, E., Lowes, M.A., Fuentes-Duculan, J., Zaba, L.C., Cardinale, I., Nograles, K.E., Khatcherian, A., Novitskaya, I., Carucci, J.A., Bergman, R., and Krueger, J.G. (2008). Low expression of the IL-23/Th17 pathway in atopic dermatitis compared to psoriasis. J. Immunol. *181*, 7420–7427.

Holtfreter, S., Radcliff, F.J., Grumann, D., Read, H., Johnson, S., Monecke, S., Ritchie, S., Clow, F., Goerke, C., Bröker, B.M., et al. (2013). Characterization of a mouse-adapted Staphylococcus aureus strain. PLoS ONE *8*, e71142.

Indra, A.K., and Leid, M. (2011). Epidermal permeability barrier measurement in mammalian skin. Methods Mol. Biol. *763*, 73–81.

Jin, H., He, R., Oyoshi, M., and Geha, R.S. (2009). Animal models of atopic dermatitis. J. Invest. Dermatol. *129*, 31–40.

Kawai, T., and Akira, S. (2010). The role of pattern-recognition receptors in innate immunity: update on Toll-like receptors. Nat. Immunol. *11*, 373–384.

Kawasaki, H., Nagao, K., Kubo, A., Hata, T., Shimizu, A., Mizuno, H., Yamada, T., and Amagai, M. (2012). Altered stratum corneum barrier and enhanced percutaneous immune responses in filaggrin-null mice. J. Allergy Clin. Immunol. *129*, 1538–46.e6.

Kim, B.S., Siracusa, M.C., Saenz, S.A., Noti, M., Monticelli, L.A., Sonnenberg, G.F., Hepworth, M.R., Van Voorhees, A.S., Comeau, M.R., and Artis, D. (2013). TSLP elicits IL-33-independent innate lymphoid cell responses to promote skin inflammation. Sci. Transl. Med. *5*, 170ra16.

Kobayashi, T., Glatz, M., Horiuchi, K., Kawasaki, H., Akiyama, H., Kaplan, D.H., Kong, H.H., Amagai, M., and Nagao, K. (2015). Dysbiosis and Staphylococcus aureus colonization drives inflammation in atopic dermatitis. Immunity *42*, 756–766.

Koga, C., Kabashima, K., Shiraishi, N., Kobayashi, M., and Tokura, Y. (2008). Possible pathogenic role of Th17 cells for atopic dermatitis. J. Invest. Dermatol. *128*, 2625–2630.

Kong, H.H., Oh, J., Deming, C., Conlan, S., Grice, E.A., Beatson, M.A., Nomicos, E., Polley, E.C., Komarow, H.D., NISC Comparative Sequence Program, et al. (2012). Temporal shifts in the skin microbiome associated with disease flares and treatment in children with atopic dermatitis. Gen. Res. *22*, 850–859.

Langley, R.G., Elewski, B.E., Lebwohl, M., Reich, K., Griffiths, C.E., Papp, K., Puig, L., Nakagawa, H., Spelman, L., Sigurgeirsson, B., et al.; ERASURE Study Group; FIXTURE Study Group (2014). Secukinumab in plaque psoriasis—results of two phase 3 trials. N. Engl. J. Med. *371*, 326–338.

Langmead, B., and Salzberg, S.L. (2012). Nmeth.1923. Nat. Methods *9*, 357–360.

Langmead, B., Trapnell, C., Pop, M., and Salzberg, S.L. (2009). Ultrafast and memory-efficient alignment of short DNA sequences to the human genome. Genome Biol. *10*, R25.

Lebwohl, M., , et al.Leonardi, C., Griffiths, C.E., Prinz, J.C., Szapary, P.O., Yeilding, N., Guzzo, C., Li, S., Hsu, M.C., Strober, B. (2011). Long-term safety experience of ustekinumab in patients with moderate-to-severe psoriasis (Part I of II): results from analyses of general safety parameters from pooled Phase 2 and 3 clinical trials. J. Am. Acad. Dermatol. *66*, 731–741.

Lee, Y., Awasthi, A., Yosef, N., Quintana, F.J., Xiao, S., Peters, A., Wu, C., Kleinewietfeld, M., Kunder, S., Hafler, D.A., et al. (2012). Induction and molecular signature of pathogenic TH17 cells. Nat. Immunol. *13*, 991–999.

Li, H., Handsaker, B., Wysoker, A., Fennell, T., Ruan, J., Homer, N., Marth, G., Abecasis, G., and Durbin, R.; 1000 Genome Project Data Processing Subgroup (2009). The Sequence Alignment/Map format and SAMtools. Bioinformatics *25*, 2078–2079.

Liu, H., Archer, N.K., Dillen, C.A., Wang, Y., Ashbaugh, A.G., Ortines, R.V., Kao, T., Lee, S.K., Cai, S.S., Miller, R.J., et al. (2017). Staphylococcus aureus epicutaneous exposure drives skin inflammation via IL-36-mediated T cell responses. Cell Host Microbe *22*, 653–666.e5.

Love, M.I., Huber, W., and Anders, S. (2014). Moderated estimation of fold change and dispersion for RNA-seq data with DESeq2. Genome Biol. *15*, 550.

Meixner, A., Zenz, R., Schonthaler, H.B., Kenner, L., Scheuch, H., Penninger, J.M., and Wagner, E.F. (2008). Epidermal JunB represses G-CSF transcription and affects haematopoiesis and bone formation. Nat. Cell Biol. *10*, 1003–1011.

Meylan, P., Lang, C., Mermoud, S., Johannsen, A., Norrenberg, S., Hohl, D., Vial, Y., Prodhom, G., Greub, G., Kypriotou, M., and Christen-Zaech, S. (2017). Skin colonization by Staphylococcus aureus precedes the clinical diagnosis of atopic dermatitis in infancy. J. Invest. Dermatol. *137*, 2497–2504.

Miyai, M., Yamamoto-Tanaka, M., Sawane, M., Yamanishi, H., Sakaguchi, M., Tsuboi, R., and Hibino, T. (2016). NOD2 inflammasome is up-regulated in the skin with atopic dermatitis. J. Dermatol. Sci. *84*, e98.

Moniaga, C.S., and Kabashima, K. (2011). Filaggrin in atopic dermatitis: flaky tail mice as a novel model for developing drug targets in atopic dermatitis. Inflamm. Allergy Drug Targets *10*, 477–485.

Naik, S., Bouladoux, N., Linehan, J.L., Han, S.J., Harrison, O.J., Wilhelm, C., Conlan, S., Himmelfarb, S., Byrd, A.L., Deming, C., et al. (2015). Commensal-dendritic-cell interaction specifies a unique protective skin immune signature. Nature *520*, 104–108.

Nair, M.G., Cochrane, D.W., and Allen, J.E. (2003). Macrophages in chronic type 2 inflammation have a novel phenotype characterized by the abundant expression of Ym1 and Fizz1 that can be partly replicated in vitro. Immunol. Lett. *85*, 173–180.

Nakagawa, S., Matsumoto, M., Katayama, Y., Oguma, R., Wakabayashi, S., Nygaard, T., Saijo, S., Inohara, N., Otto, M., Matsue, H., et al. (2017). Staphylococcus aureus virulent PSMα peptides induce keratinocyte alarmin release to orchestrate IL-17-dependent skin inflammation. Cell Host Microbe *22*, 667–677.e5.

ORegan, G.M., Sandilands, A., McLean, W.H., and Irvine, A.D. (2009). Filaggrin in atopic dermatitis. J. Allergy Clin. Immunol. *124*, R2–R6.

Olsson, M., Broberg, A., Jernås, M., Carlsson, L., Rudemo, M., Suurküla, M., Svensson, P.A., and Benson, M. (2006). Increased expression of aquaporin 3 in atopic eczema. Allergy *61*, 1132–1137.

Oyoshi, M.K., Murphy, G.F., and Geha, R.S. (2009). Filaggrin-deficient mice exhibit TH17-dominated skin inflammation and permissiveness to epicutaneous sensitization with protein antigen. J. Allergy Clin. Immunol. *124*, 485–493.e1.

Paller, A.S., Kabashima, K., and Bieber, T. (2017). Therapeutic pipeline for atopic dermatitis: End of the drought? J. Allergy Clin. Immunol. *140*, 633–643.

Pflegerl, P., Vesely, P., Hantusch, B., Schlederer, M., Zenz, R., Janig, E., Steiner, G., Meixner, A., Petzelbauer, P., Wolf, P., et al. (2009). Epidermal loss of JunB leads to a SLE phenotype due to hyper IL-6 signaling. Proc. Natl. Acad. Sci. U S A *106*, 20423–20428.

Rak, G.D., Osborne, L.C., Siracusa, M.C., Kim, B.S., Wang, K., Bayat, A., Artis, D., and Volk, S.W. (2016). IL-33-dependent group 2 innate lymphoid cells promote cutaneous wound healing. J. Invest. Dermatol. *136*, 487–496.

Rankin, L.C., Girard-Madoux, M.J., Seillet, C., Mielke, L.A., Kerdiles, Y., Fenis, A., Wieduwild, E., Putoczki, T., Mondot, S., Lantz, O., et al. (2016). Complementarity and redundancy of IL-22-producing innate lymphoid cells. Nat. Immunol. *17*, 179–186.

Ratner, M. (2015). IL-17-targeting biologics aim to become standard of care in psoriasis. Nat. Biotechnol. *33*, 3–4.

Roediger, B., Kyle, R., Yip, K.H., Sumaria, N., Guy, T.V., Kim, B.S., Mitchell, A.J., Tay, S.S., Jain, R., Forbes-Blom, E., et al. (2013). Cutaneous immunosurveillance and regulation of inflammation by group 2 innate lymphoid cells. Nat. Immunol. *14*, 564–573.

Salimi, M., Barlow, J.L., Saunders, S.P., Xue, L., Gutowska-Owsiak, D., Wang, X., Huang, L.C., Johnson, D., Scanlon, S.T., McKenzie, A.N., et al. (2013). A role for IL-25 and IL-33-driven type-2 innate lymphoid cells in atopic dermatitis. J. Exp. Med. *210*, 2939–2950.

Schmitz, A., Lazić, E., Koumaki, D., Kuonen, F., Verykiou, S., and Rübsam, M. (2015). Assessing the in vivo epidermal barrier in mice: dye penetration assays. J. Invest. Dermatol. *135*, 1–4.

Simpson, E.L., Bieber, T., Guttman-Yassky, E., Beck, L.A., Blauvelt, A., Cork, M.J., Silverberg, J.I., Deleuran, M., Kataoka, Y., Lacour, J.P., et al.; SOLO 1 and SOLO 2 Investigators (2016). Two phase 3 trials of dupilumab versus placebo in atopic dermatitis. N. Engl. J. Med. *375*, 2335–2348.

Singh, K., Camera, E., Krug, L., Basu, A., Pandey, R.K., Munir, S., Wlaschek, M., Kochanek, S., Schorpp-Kistner, M., Picardo, M., et al. (2018). JunB defines functional and structural integrity of the epidermo-pilosebaceous unit in the skin. Nat. Commun. *9*, 3425.

Spits, H., Artis, D., Colonna, M., Diefenbach, A., Di Santo, J.P., Eberl, G., Koyasu, S., Locksley, R.M., McKenzie, A.N., Mebius, R.E., et al. (2013). Innate lymphoid cells—a proposal for uniform nomenclature. Nat. Rev. Immunol. *13*, 145–149.

Subramanian, A., Tamayo, P., Mootha, V.K., Mukherjee, S., Ebert, B.L., Gillette, M.A., Paulovich, A., Pomeroy, S.L., Golub, T.R., Lander, E.S., and Mesirov, J.P. (2005). Gene set enrichment analysis: a knowledge-based approach for interpreting genome-wide expression profiles. Proc. Natl. Acad. Sci. U S A *102*, 15545–15550.

Sumaria, N., Roediger, B., Ng, L.G., Qin, J., Pinto, R., Cavanagh, L.L., Shklovskaya, E., Fazekas de St Groth, B., Triccas, J.A., and Weninger, W. (2011). Cutaneous immunosurveillance by self-renewing dermal gammadelta T cells. J. Exp. Med. *208*, 505–518.

Trapnell, C., Hendrickson, D.G., Sauvageau, M., Goff, L., Rinn, J.L., and Pachter, L. (2013). Differential analysis of gene regulation at transcript resolution with RNA-seq. Nat Biotechnol. *31*, 46–53.

Tsoi, L.C., Rodriguez, E., Degenhardt, F., Baurecht, H., Wehkamp, U., Volks, N., Szymczak, S., Swindell, W.R., Sarkar, M.K., Raja, K., et al. (2019). Atopic dermatitis is an IL-13-dominant disease with greater molecular heterogeneity compared to psoriasis. J. Invest. Dermatol. *139*, 1480–1489.

Turner, M.J., and Zhou, B. (2014). A new itch to scratch for TSLP. Trends Immunol. *35*, 49–50.

Uluçkan, Ö., and Wagner, E.F. (2016). Role of IL-17A signalling in psoriasis and associated bone loss. Clin. Exp. Rheumatol. *34*, 17–20.

Uluçkan, Ö., and Wagner, E.F. (2017). Chronic systemic inflammation originating from epithelial tissues. FEBS J. *284*, 505–516.

Uluçkan, Ö., Jimenez, M., Karbach, S., Jeschke, A., Graña, O., Keller, J., Busse, B., Croxford, A.L., Finzel, S., Koenders, M., et al. (2016). Chronic skin inflammation leads to bone loss by IL-17-mediated inhibition of Wnt signaling in osteoblasts. Sci. Transl. Med. *8*, 330ra37.

Weidinger, S., Beck, L.A., Bieber, T., Kabashima, K., and Irvine, A.D. (2018). Atopic dermatitis. Nat. Rev. Dis. Primers *4*, 1.

Welch, J.S., Escoubet-Lozach, L., Sykes, D.B., Liddiard, K., Greaves, D.R., and Glass, C.K. (2002). TH2 cytokines and allergic challenge induce Ym1 expression in macrophages by a STAT6-dependent mechanism. J. Biol. Chem. *277*, 42821–42829.

Werfel, T., Allam, J.P., Biedermann, T., Eyerich, K., Gilles, S., Guttman-Yassky, E., Hoetzenecker, W., Knol, E., Simon, H.U., Wollenberg, A., et al. (2016). Cellular and molecular immunologic mechanisms in patients with atopic dermatitis. J. Allergy Clin. Immunol. *138*, 336–349.

Williams, M.R., Nakatsuji, T., and Gallo, R.L. (2017). Staphylococcus aureus: Master Manipulator of the Skin. Cell Host Microbe *22*, 579–581.

Wilson, S.R., Thé, L., Batia, L.M., Beattie, K., Katibah, G.E., McClain, S.P., Pellegrino, M., Estandian, D.M., and Bautista, D.M. (2013). The epithelial cell-derived atopic dermatitis cytokine TSLP activates neurons to induce itch. Cell *155*, 285–295.

Woodring, T., Kobayashi, T., Kim, D., and Nagao, K. (2018). ADAM17-deficient mice model the transcriptional signature of human atopic dermatitis. J. Invest. Dermatol. *138*, 2283–2286.

STAR★METHODS

KEY RESOURCES TABLE

REAGENT or RESOURCE	SOURCE	IDENTIFIER
Antibodies		
S. aureus antibody	Abcam	Cat#ab20920; RRID: AB_445913
Loricrin antibody	Covance	Cat#PRB-145P; RRID: AB_10064155
MyD88 antibody	Cell Signaling	Cat#D80F5; RRID: AB_10547882
CD45-APCCy7	Biolegend	Cat#103115; RRID: AB_312980
CD3-PE	Biolegend	Cat#100205; RRID: AB_312662
gdTCR-BV421	Biolegend	Cat#118119; RRID: AB_10896753
CD90.2-APC	BD PharMingen	Cat#553007; RRID: AB_398526
CD11b-PerCPCy5.5	BD PharMingen	Cat#550993; RRID: AB_394002
CD2-PeCy7	Biolegend	Cat#100113; RRID: AB_2563091
MHCII-Pacific Blue	Biolegend	Cat#107619; RRID: AB_493528
Ly6C-APC	BD Biosciences	Cat#560595; RRID: AB_1727554
Ly6G-PE	BD Biosciences	Cat#561104; RRID: AB_10563079
CD11b-PeCy7	BD PharMingen	Cat#552850; RRID: AB_394491
Chemicals, Peptides, and Recombinant Proteins		
Aldara 5% cream	Meda	N/A
Streptavidin-AF488	Invitrogen	S11223
Deposited Data		
RNaseq data from skin from control and JunB$^{\Delta ep}$ mice	This paper	GSE136657
Experimental Models: Organisms/Strains		
Mouse: JunBΔep: B6.129P2-Junbtm3Wag/J crossed to K5-Cre	N/A	N/A
Mouse: Rag1−/−: B6.129S7-*Rag1*^{tm1Mom}/J	N/A	N/A
Mouse: IL-17A-eGFP: C57BL/6-*Il17a*^{tm1Bcgen}/J	N/A	N/A
Oligonucleotides		
Oligos for q-RT-PCR	See Table S1 for oligo sequences	N/A
Software and Algorithms		
Nextpresso	Graña et al., 2018	http://bioinfo.cnio.es/nextpresso/
FastQC (v 0.11.0)		https://www.bioinformatics.babraham.ac.uk/projects/fastqc/
Bowtie (v 1.0.0 70)	Langmead and Salzberg, 2012	http://bowtie-bio.sourceforge.net/bowtie2/index.shtml
SAMtools (v 0.1.19 71)	Li et al., 2009	http://samtools.sourceforge.net/
GSEA v(2.08)	Subramanian et al., 2005	http://software.broadinstitute.org/gsea/index.jsp
TopHat (v 2.0.10 69)	Kim et al., 2013	https://ccb.jhu.edu/software/tophat/index.shtml

LEAD CONTACT AND MATERIALS AVAILABILITY

Further information may be directed to and will be fulfilled by the Lead Contact, Özge Uluçkan (ozge.uluckan@novartis.com). This study did not generate new unique reagents.

EXPERIMENTAL MODEL AND SUBJECT DETAILS

Mice

All mouse experiments were performed in accordance with local and institutional regulations/licenses. The generation of the *JunB*$^{\Delta ep}$ mice has previously been described (Meixner et al., 2008). *JunB*$^{\Delta ep}$ mice were backcrossed to C57B/6 for at least 7 generations. Mice

of both sexes were included in the study, since no gender differences were observed. *Il17a*-eGFP (C57BL/6-*Il17a*$^{tm1Bcgen/J}$) mice, originally generated at Biocytogen and previously described (Lee et al., 2012), were purchased from Jackson Laboratory.

METHOD DETAILS

Histological analyses

Tissues were fixed in phosphate-buffered saline–buffered 3.7% formalin. H&E staining was performed according to standard procedures (Sigma). Immunohistochemistry was performed with Elite ABC Kit (Vectastain) and DAB (Vector Laboratories). Immunofluorescence was performed as described previously (Guinea-Viniegra et al., 2014). In short, tissues were fixed in phosphate-buffered saline–buffered 3.7% formalin. Primary antibodies were incubated overnight at 4C and secondary antibodies for 1 hour at RT. Counterstainings were performed with 4′,6-diamidino-2-phenylindole (Sigma) and sections were mounted using Prolong Gold anti-fade reagent (ThermoFisher).

Gram staining was carried out using provider's instructions (Sigma). Toluidine Blue staining was performed using standard protocols. *S. aureus* antibody was purchased from Abcam (ab20920), Loricrin and Keratin 6 antibodies from Covance (PRB-145P), and MyD88 antibody (D80F5) from Cell Signaling. Secondary antibodies were purchased from Invitrogen and used at 1:250 dilution.

Barrier analysis

Trans-epidermal water loss was measured using Tewameter TM300 from Courage-Khazaka.

Outside-in Toludine blue assay was carried out as described in Indra and Leid (2011) and briefly embryos isolated at embryonic day E17.5 were incubated in methanol for 5 mins, rinsed in PBS, then incubated in 0.1% Toluidine Blue for 5 mins. Embryos were kept in PBS and photographed immediately afterward.

Inside-out biotin diffusion assay was carried out as described in Schmitz et al. (2015). 10 mg/ml EZ-Link sulfo-NHS-LC-biotin (Pierce Chemical Co., Rockford, IL) in PBS containing 1 mM $CaCl_2$ was injected intradermally into the back skin of 1-day old mice. After 30 minutes, mice were sacrificed and the skin was embedded in Tissue-Tek and snap-frozen on dry ice. Paraformaldehyde-fixed cryosections were stained with Occludin antibody (Thermo-Fisher-Zymed (40-6100)). Sections were then stained with streptavidin-AF488 (Invitrogen), a AF555-labeled secondary antibody to detect occludin and DAPI to visualize the nuclei.

Flow cytometry

Skin from mice was isolated and subjected to mild digestion with liberase (Roche^{TM}), and mechanically disrupted using GentleMACS Dissociator (Miltenyi). The single cell suspension was stained with either CD45-APCCy7 (Biolegend), CD3-PE (Biolegend), gdTCR-Brilliant Violet 421 (Biolegend), CD90.2-APC (BD PharMingen), Cd11b-PerCPCy5.5 (BD PharMingen) and CD2-PECy7 (Biolegend), or CD45-APCCy7 (Biolegend), MHCII-PacBlue (Biolegend), Ly6C-APC (BD PharMingen), Ly6G-PE (BD PharMingen), CD11b-PECy7 (BD PharMingen). Samples were collected in a FACS CANTO II (BD, San Jose CA) equipped with 488nm, 640nm and 405nm lines. We used pulse processing to exclude cell aggregates and live/dead fixable dye Aqua (Invitrogen) to exclude dead cells. At least 100,000 alive single events were collected; all data were analyzed using FlowJo v10 (Treestar, Oregon).

Antibiotic administration

Enrofloxacin (Baytril) (0.025%) was added to the drinking water either at weaning (for prophylactic experiments) or at 6 months of age for 1, 2 or 3 weeks and changed every 3 days. For the prophylactic experiments, enrofloxacin was kept in the drinking water thoughout the lifetime of the mice.

Bacterial analysis

Columbia agar +5% Sheep blood plates and Mannitol-salt agar plates were purchased from Biomerieux (43041, 43671). Swabs were dipped in PBS, lesional skin of mice were swabbed and the swabs were directly inoculated on the plates. Plates were incubated at 37C for 24 hours. DNA was isolated from single colonies grown on blood agar plates, and PCR was carried. Oligo sequences are included in Table S1. USA300 *S. aureus* strain and a specimen from a nearby hospital were used as positive controls. To measure CFUs of *S. aureus*, we performed a standard plate count method. Briefly, skin swabs from the lesional skin of *JunB*$^{\Delta ep}$ mice (1 cm^2), and equivalent area in control mice were placed into 1 mL of PBS, and serial dilutions were prepared. 200 μL of each dilution was transferred on blood agar plates, and colonies were counted after a 1-day incubation. CFUs were calculated by dividing the number of colonies per plate by the dilution factor.

Transcriptomic analysis

Samples of 1ug of total RNA were used. Average sample RNA integrity number was 7.75 (range 7-9.2) when assayed on Agilent 2100 Bioanalyzer. PolyA+ fraction was purified and randomly fragmented, converted to double stranded cDNA and processed through subsequent enzymatic treatments of end-repair, dA-tailing, and ligation to adapters as in Illumina's "TruSeq Stranded mRNA Sample Preparation Part # 15031047 Rev. D" kit (this kit incorporates dUTP during 2nd strand cDNA synthesis, which implies that only the cDNA strand generated during 1st strand syntesis is eventually sequenced). Adaptor-ligated library was completed by PCR with Illumina PE primers. The resulting purified cDNA library was applied to an Illumina flow cell for cluster

generation and sequenced on an Illumina instrument (see below) by following manufacturer's protocol. Sequencing reads were analyzed with the next*presso* pipeline (Graña et al., 2018), as follows: sequencing quality was checked with FastQC v0.11.0 (http://www.bioinformatics.babraham.ac.uk/projects/fastqc/). Reads were aligned to the mouse genome (NCBI37/mm9) with TopHat-2.0.10 (Trapnell et al., 2013) using Bowtie 1.0.0 (Langmead et al., 2009) and SAMtools 0.1.19 (Li et al., 2009), allowing 2 mismatches and 20 multihits. Differential expression was tested with DESeq2 (Love et al., 2014), using the mouse NCBI37/mm9 transcript annotations from https://ccb.jhu.edu/software/tophat/igenomes.shtml. GSEAPreranked (Subramanian et al., 2005) was used to perform gene set enrichment analysis of the described gene signatures on a pre-ranked gene list, setting 1000 gene set permutations.

QUANTIFICATION AND STATISTICAL ANALYSIS

Two-tailed students's t test after Shapiro-Wilk normality test was used to determine statistical significance. Linear regression analyses was used for the correlation study in Figure 2E. One-way ANOVA with multiple-comparisons was used for Figures 4C and 6B and 7C.

DATA AND CODE AVAILABILITY

Data from RNA sequencing analysis has been deposited to GEO with accession number GEO: GSE136657.

Cell Reports, Volume *29*

Supplemental Information

Cutaneous Immune Cell-Microbiota Interactions Are Controlled by Epidermal JunB/AP-1

Özge Uluçkan, Maria Jiménez, Ben Roediger, Jakob Schnabl, Lucía T. Díez-Córdova, Kevin Troulé, Wolfgang Weninger, and Erwin F. Wagner

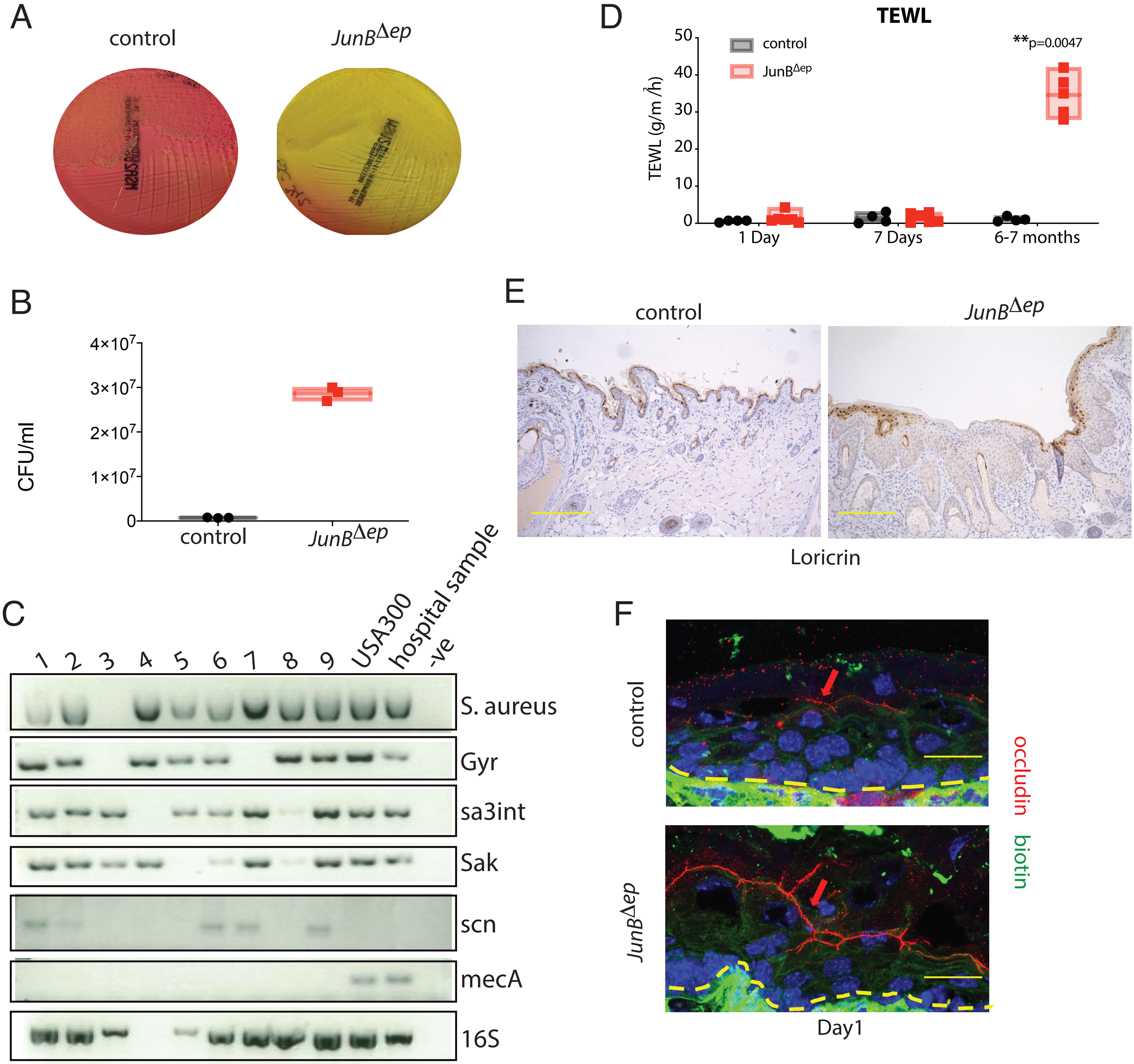

Figure S1. *JunB*$^{\Delta ep}$ mice have human-derived *S. aureus* colonization and transient barrier defects. Related to Figure 1. A) Growth of bacteria on mannitol-salt agar plates isolated from control vs *JunB*$^{\Delta ep}$ skin (n>10 per genotype). B) CFUs/ml of bacteria isolated control versus *JunB*$^{\Delta ep}$skin. C) PCR for S. aureus, S. aureus specific gyrase (gyr), human-specific immune invasion gene cluster including Staphylokinase (sak) and staphylococcal complement inhibitor (scn), a human-specific prophase integrase-gene sa3int and methicillin-resistance gene mecA with 16S as control from from single colonies of bacteria grown on blood agar plates from *JunB*$^{\Delta ep}$ skin. D) Trans-epidermal water loss measurements 1 and 7 days post-birth of control and *JunB*$^{\Delta ep}$ mice, as well as 6-7 months old control and *JunB*$^{\Delta ep}$ mice (n=4,6) (lesional skin). E) Loricrin staining at 6-7 months of age of the lesional skin of control and *JunB*$^{\Delta ep}$ mice (n=5,6). Yellow scale bars indicate 100μm. F) Inside-out barrier assay using biotin diffusion in control and *JunB*$^{\Delta ep}$ mice at 1-day post birth (n=4,5). Biotin-streptavidin is shown in green and occludin IF is shown in red.
Dashed yellow line indicates the basement membrane separating epidermis-dermis.
Red arrows show where the green biotin staining ends. Yellow scale bars indicate 50μm.

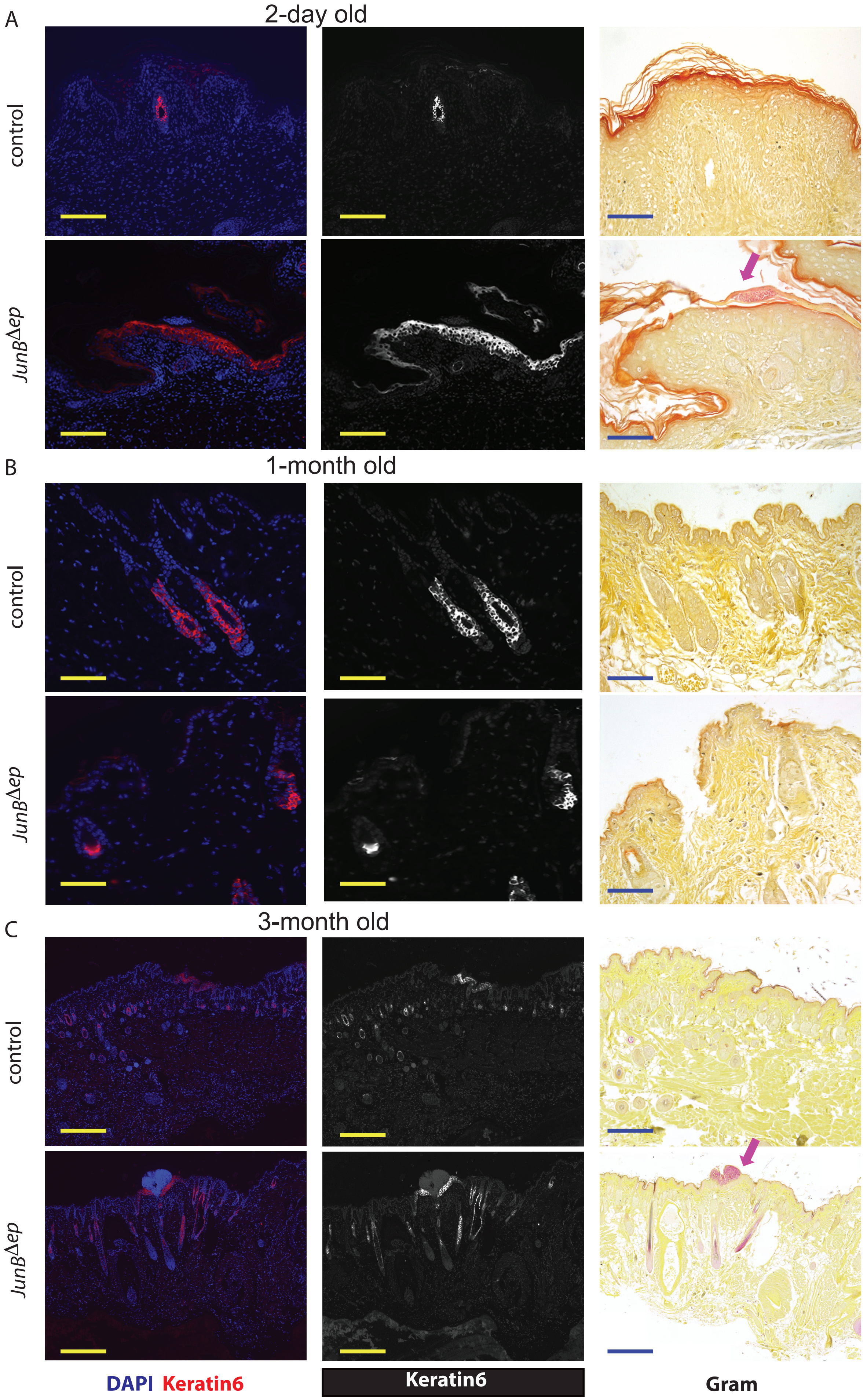

Figure S2. *JunB*$^{\Delta ep}$ mice do not have S. aureus colonization early in life. Related to Figure 1. Keratin6 and Gram staining in skin from control and *JunB*$^{\Delta ep}$ mice at 2 days (n=4,5), 1 month (n=4,4) and 3 months (n=7,8) post-birth. The yellow scale bars indicate 200μm in A, 100μm B and 500μm in C. The blue scale bars indicate 100μm in A, B and 500μm in C.

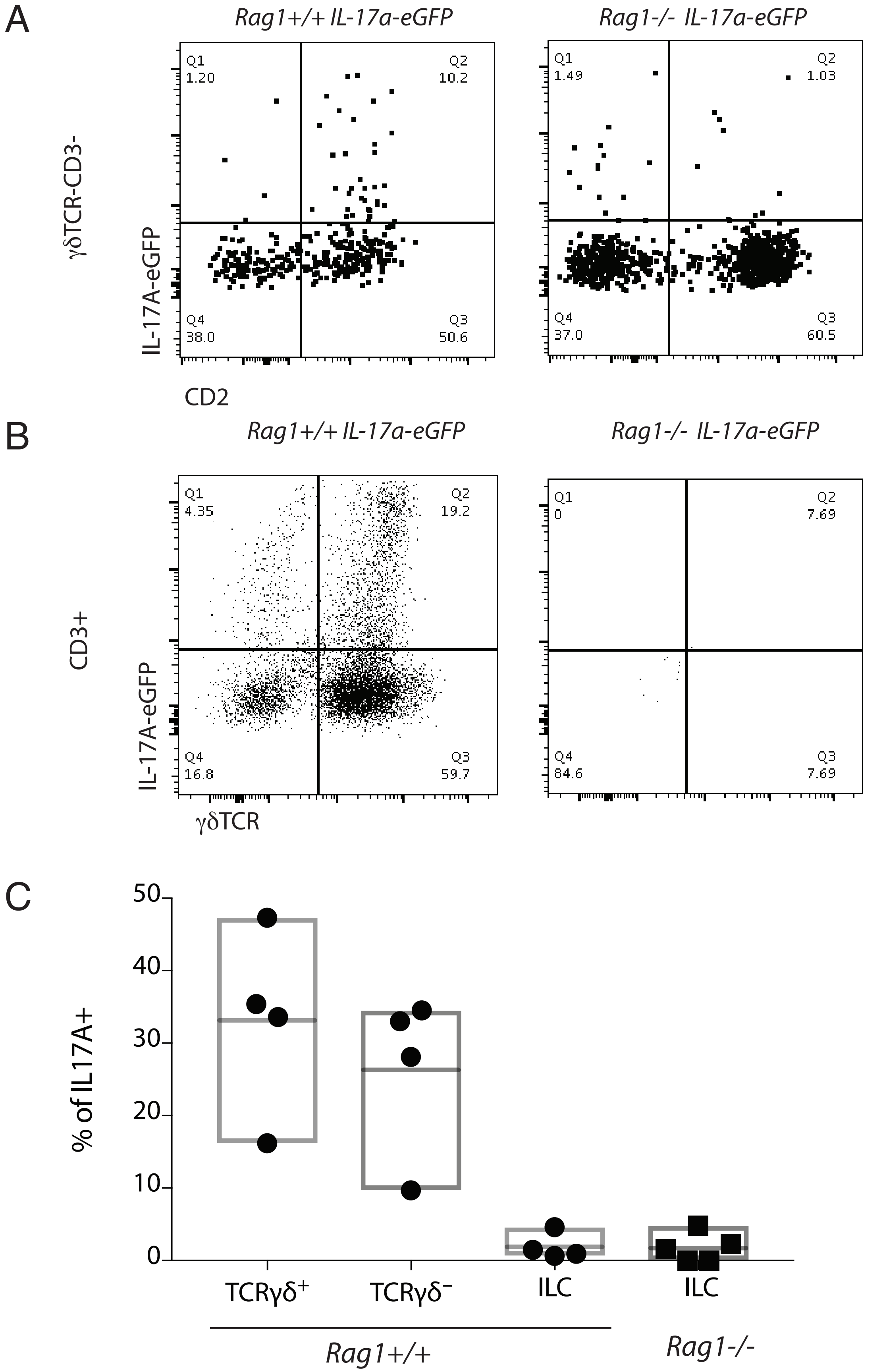

Figure S3. ILC3s do not compensate for IL-17A production by T-cells in *Rag1-/-* mice upon IMQ administration. Related to Figure 4.

A) Representative flow cytometry images of IL-17A expressing ILCs of control and *Rag1-/-* mice upon 5-days of IMQ administration on the ears (n=4,5). B) Representative flow cytometry images of IL-17A expressing T-cells of control and *Rag1-/-* mice 5-days of IMQ administration on the ears. C) Quantification of the distribution of IL-17A+ cells in *Rag1+/+* compared to *Rag1-/-* mice.

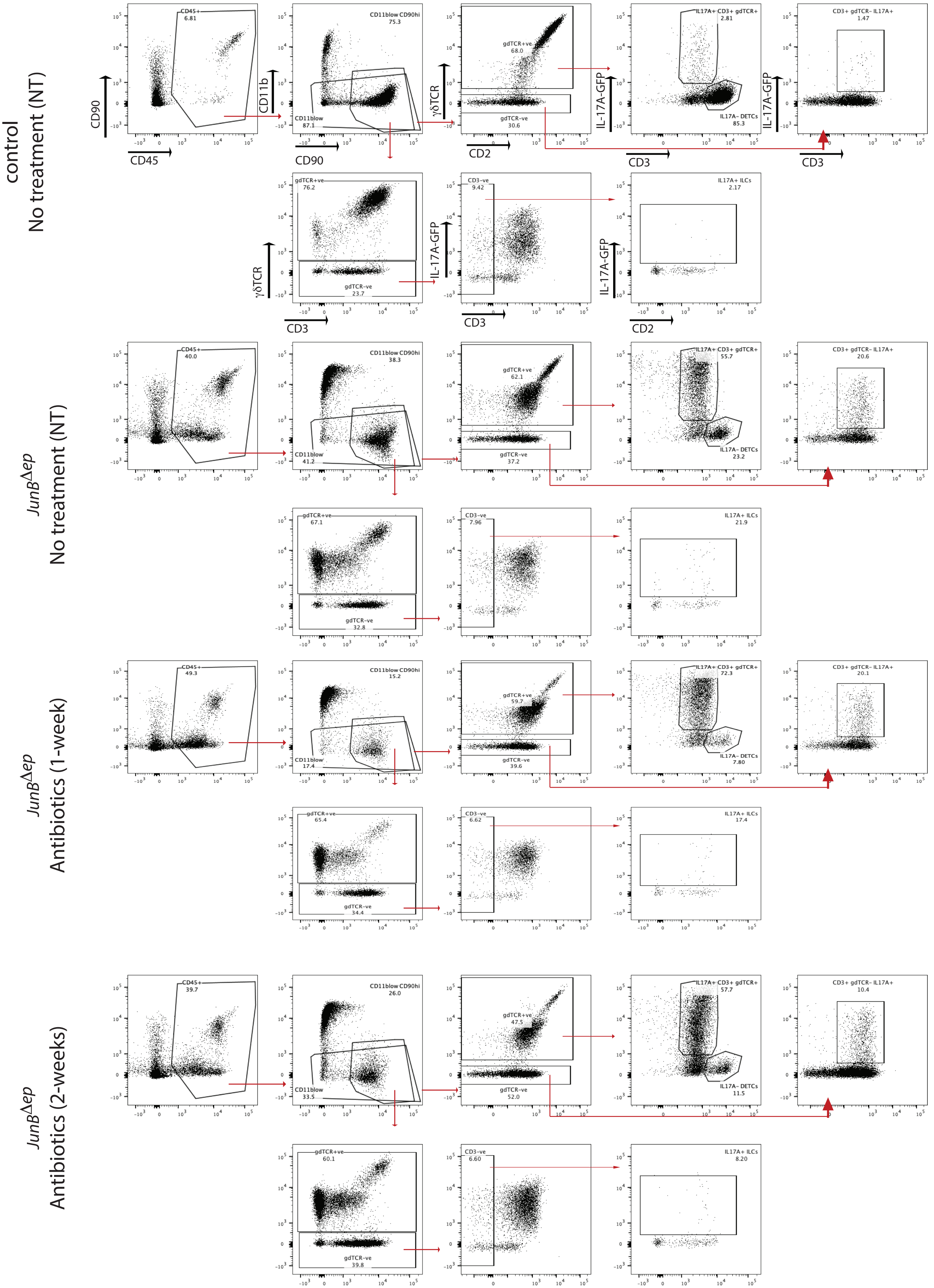

Figure S4. Antibiotic treatment post development of skin inflammation does not provide therapeutic benefit. Related to Figure 6.

Conventional flow plots (including gating strategy) of lymphoid cells in *JunB*$^{\Delta ep}$ mice with or without 1 and 2-weeks of antibiotics compared to controls.

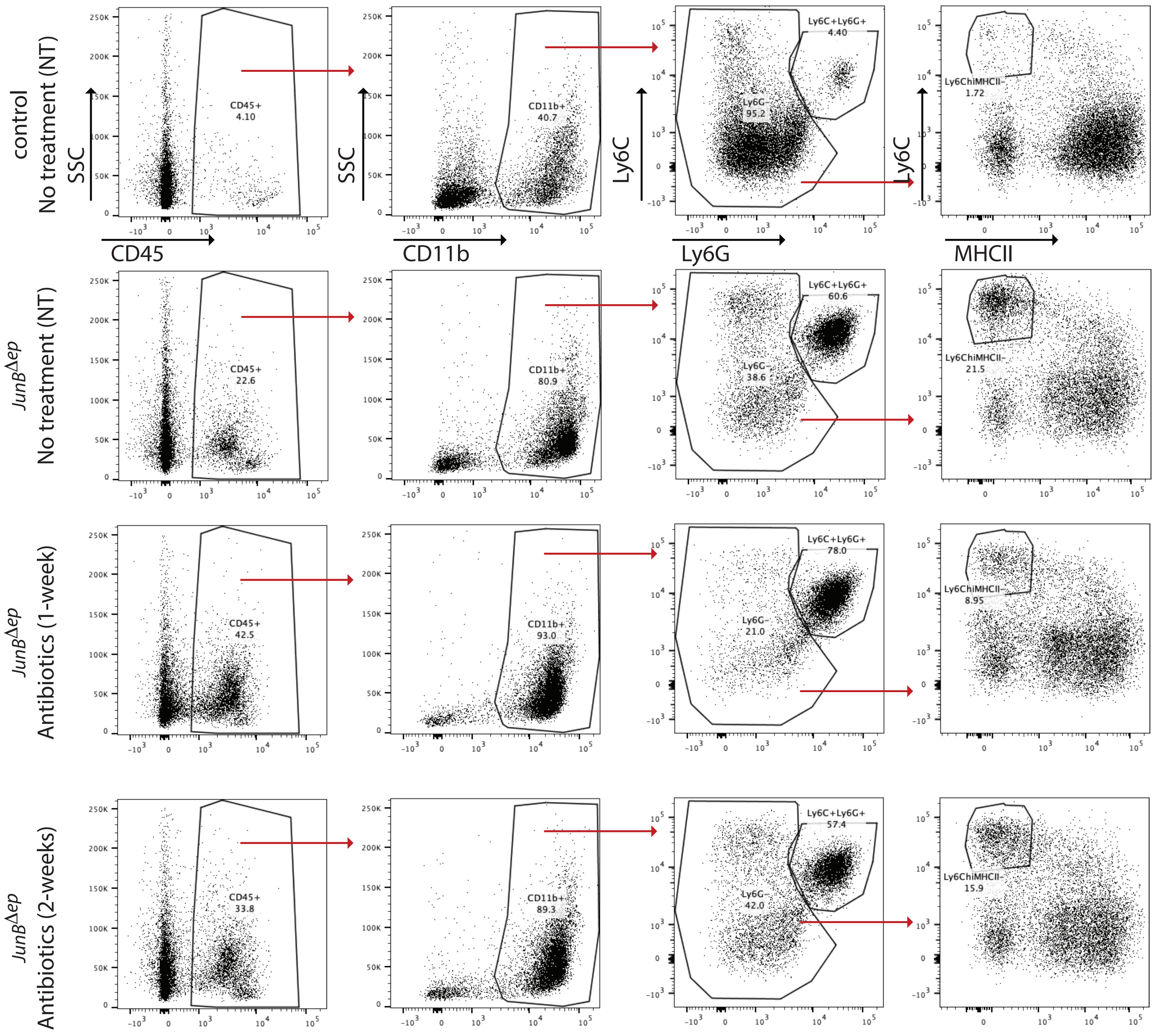

Figure S5. Antibiotic treatment post development of skin inflammation does not provide therapeutic benefit. Related to Figure 6.

Conventional flow plots (including gating strategy) of myeloid cells in *JunB*$^{\Delta ep}$ mice with or without 1 and 2-weeks of antibiotics compared to controls.

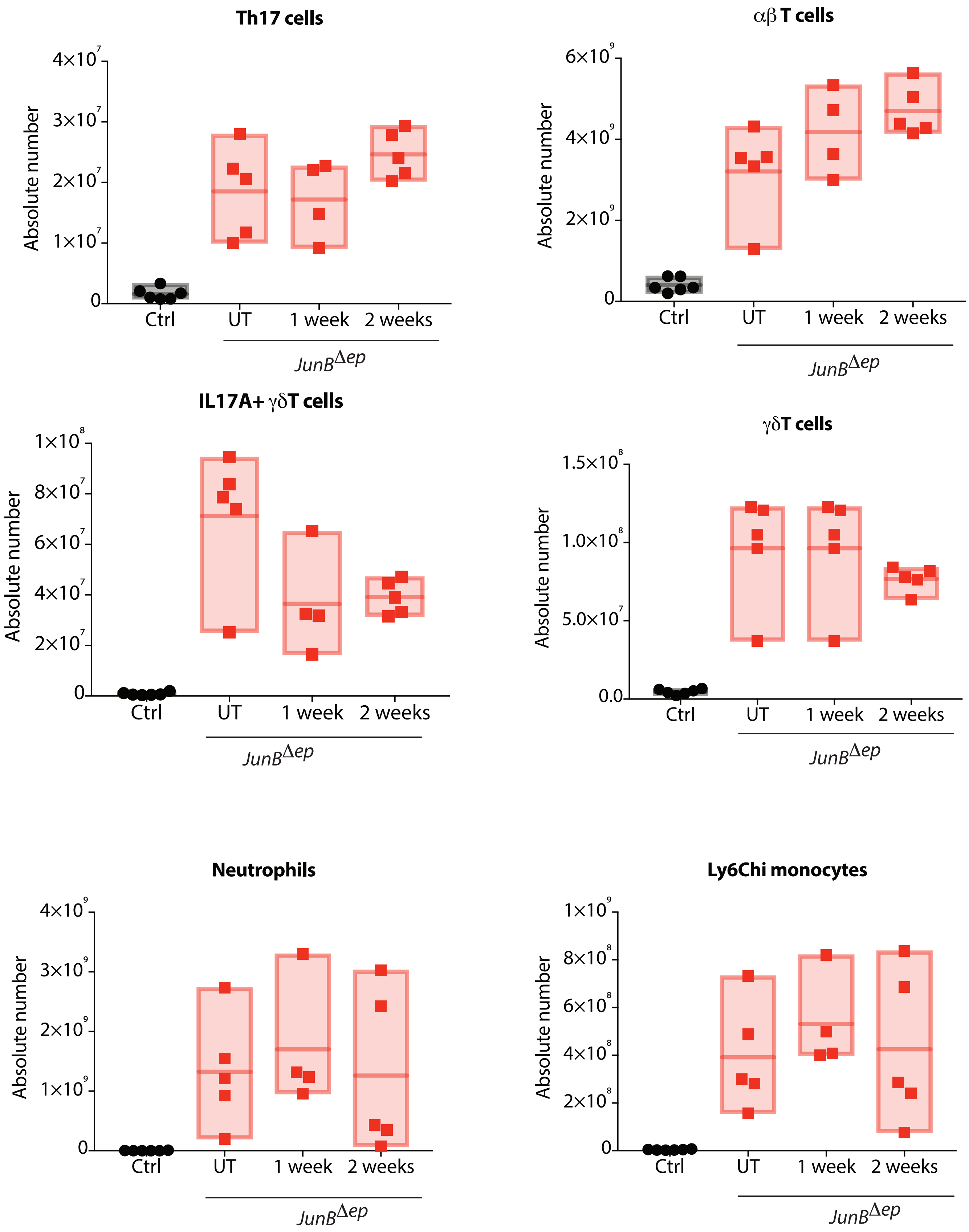

Figure S6. Increased number of γδTcells in lymph nodes upon 2-weeks of antibiotic treatment. Related to Figure 6. Number of infiltrating immune cells into the skin-draining lymph nodes of *JunB*$^{\Delta ep}$ mice with or without 1 and 2-weeks of antibiotics compared to controls as analysed by flow cytometry.

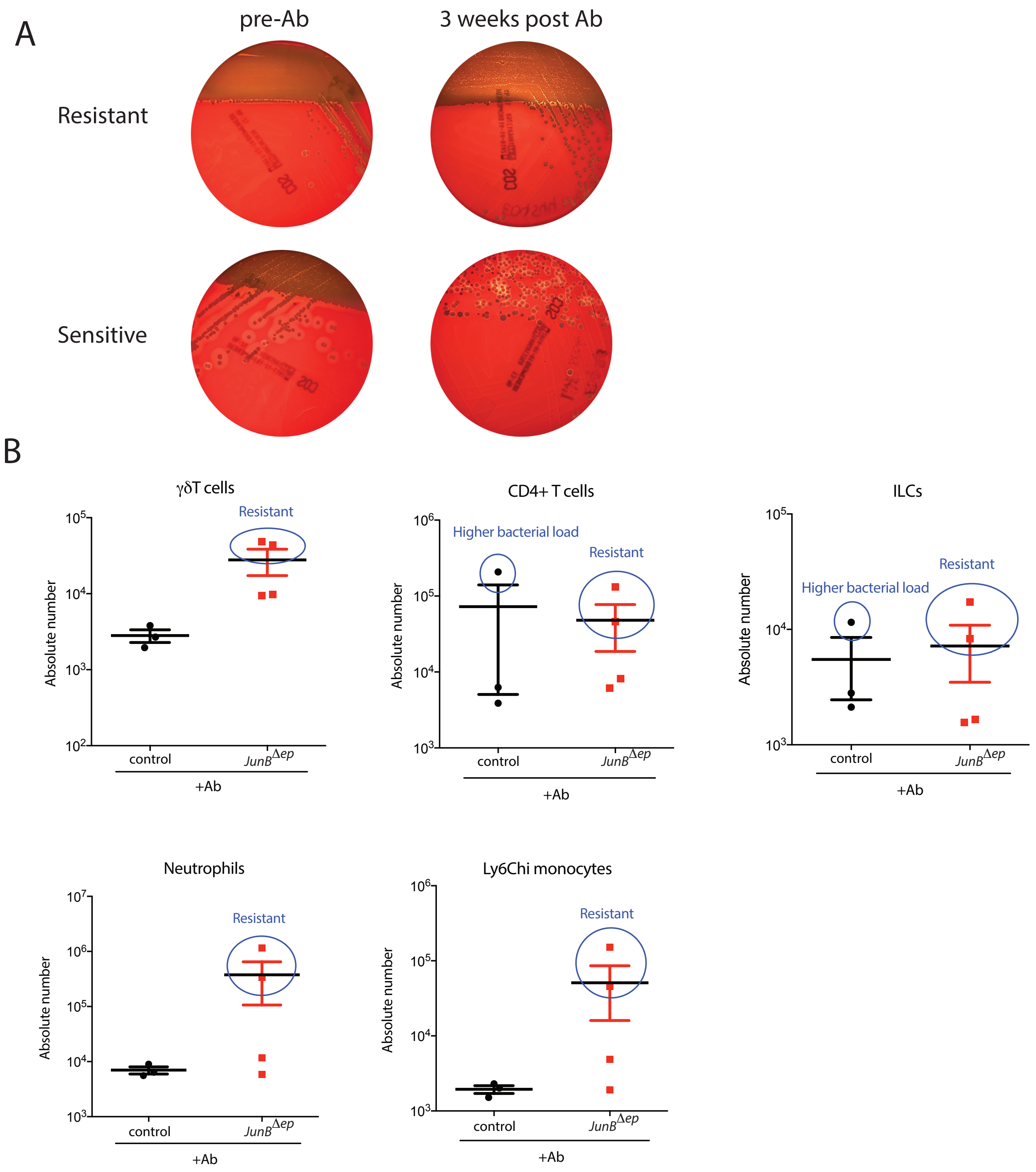

Figure S7. Characterization of the immune cells in mice resistant to antibiotics. Related to Figure 6.
A) Representative images of blood agar plates of swabs derived from *JunB*$^{\Delta ep}$ mice skin sensitive or resistant to 3-weeks of antibiotic treatment post-lesion development.
B) Number of immune cell infiltrates into the skin of *JunB*$^{\Delta ep}$ mice skin sensitive or resistant to 3-weeks of antibiotic treatment post-lesion development

Name	F (5'-3')	R (5'-3')
**Duox1**	GATTCTCCTGGTTGGGACA	CCTGCAGAACCTCTCATGA
**Il13ra2**	AGCGAATGGAGTGAAGAGGA	GCTCAATGTGGGTTCAGGTT
**Stac2**	CAAGCTCCAGCGATTTAAGCG	AGCCTCACTGGTTTGAGCG
**Tslp**	ACGGATGGGGCTAACTTACAA	AGTCCTCGATTTGCTCGAACT
**Aqp3**	AACCCTGCTGTGACCTTTG	GCTGCTGTGCCTATGAACTG
**Il4ra**	TGGGCTGTCGATTTTGCTTTTGG	GTGCTGGGGTGGGAATCTGGTC
**Nod2**	CAGGTCTCCGAGAGGGTACTG	GCTACGGATGAGCCAAATGAAG
**Osm**	TGCCCGGCACAATATCCTC	GTGTGGGCTCAGGTATCTCCAG
**Slc4a11**	CAGGACTCCGGTGAATACTTCT	GATGCTCTCGCCAGACACAA
**Mmp13**	CTTCTTCTTGTTGAGCTGGACTC	CTGTGGAGGTCACTGTAGACT
**Serpinb3a**	CAGATGATGAAACAAAACATCG	AGACCTTGAGTGCTGCTCATA
**il4**	GGTCTCAACCCCCAGCTAGT	GCCGATGATCTCTCTCAAGTGAT
**il1b**	GCAACTGTTCCTGAACTCAACT	ATCTTTTGGGGTCCGTCAACT
**lcn2**	TGGCCCTGAGTGTCATGTG	CTCTTGTAGCTCATAGATGGTGC
**S100a8**	AAATCACCATGCCCTCTACAAG	CCCACTTTTATCACCATCGCAA
**mdefb14**	TCCAGGGGACGCATTCCTA	ACCGCTATTAGAACATCGACCTA
**il36a (il1f6)**	GCAGCATCACCTTCGCTTAGA	CAGATATTGGCATGGGAGCAAG
**il36b (il1f8)**	AGAGTATTCAAATGTGGGAACCG	GACCCATACCATCTGTTGTGAG
**il36g (il1f9)**	TCCTGACTTTGGGGAGGTTTT	TCACGCTGACTGGGGTTACT
**cxcl1**	CTGGGATTCACCTCAAGAACATC	CAGGGTCAAGGCAAGCCTC
**cxcl2**	CCAACCACCAGGCTACAGG	GCGTCACACTCAAGCTCTG
**ccl2**	TTAAAAACCTGGATCGGAACCAA	GCATTAGCTTCAGATTTACGGGT
**S100a9**	ATACTCTAGGAAGGAAGGACACC	TCCATGATGTCATTTATGAGGGC
**S.aureus**	AATCTTTGTCGGTACACGATATTCTTCACG	CGTAATGAGATTTCAGTAGATAATACAACA
**Gyr**	AGTACATCGTCGTATACTATATGG	ATCACGTAACAGTTCAAGTGTG
**sa3int**	GAAAAACAAACGGTGCTAT	TTATTGACTCTACAGGCTGA
**Sak**	AAGGCGATGACGCGAGTTAT	GCGCTTGGATCTAATTCAAC
**scn**	AGCACAAGCTTGCCAACATCG	TTAATATTTACTTTTTAGTGC
**mecA**	AAAATCGATGGTAAAGGTTGGC	AGTTCTGCAGTACCGGATTTGC
**16S**	9AACTCTGTTATTAGGGAAGAACA	CCACCTTCCTCCGGTTTGTCACC

Table S1. Sequences of Oligonucleotides. Related to STAR Methods.
